# Fabrication of Smart Tantalum Carbide MXene Quantum Dots with Intrinsic Immunomodulatory Properties for Treatment of Allograft Vasculopathy

**DOI:** 10.1002/adfm.202106786

**Published:** 2021-09-08

**Authors:** Alireza Rafieerad, Weiang Yan, Keshav Narayan Alagarsamy, Abhay Srivastava, Niketa Sareen, Rakesh C. Arora, Sanjiv Dhingra

**Affiliations:** ^1^ Regenerative Medicine Program Department of Physiology and Pathophysiology Rady Faculty of Health Sciences University of Manitoba Winnipeg Manitoba R3E 0W2 Canada; ^2^ Institute of Cardiovascular Sciences Albrechtsen St. Boniface Research Centre University of Manitoba Winnipeg Manitoba R2H 2A6 Canada; ^3^ Section of Cardiac Surgery Department of Surgery Max Rady College of Medicine Rady Faculty of Health Sciences University of Manitoba Winnipeg Manitoba R3E 0W2 Canada

**Keywords:** allograft vasculopathy, bioactive material, hydrofluoric acid‐free synthesis, in vivo immunomodulation, Ta _4_C _3_T*_x_* MXene quantum dots

## Abstract

MXene nanomaterials have sparked significant interest among interdisciplinary researchers to tackle today's medical challenges. In particular, colloidal MXene quantum dots (MQDs) offer the high specific surface area and compositional flexibility of MXene while providing improvements to aqueous stability and material–cell interactions. The current study for the first time reports the development and application of immunoengineered tantalum‐carbide (Ta_4_C_3_T*_x_*) MQDs for in vivo treatment of transplant vasculopathy. This report comes at a critical juncture in the field as poor long‐term safety of other MXene compositions challenge the eventual clinical translatability of these materials. Using rational design and synthesis strategies, the Ta_4_C_3_T*_x_* MQDs leverage the intrinsic anti‐inflammatory and antiapoptotic properties of tantalum to provide a novel nanoplatform for biomedical engineering. In particular, these MQDs are synthesized with high efficiency and purity using a facile hydrofluoric acid‐free protocol and are enriched with different bioactive functional groups and stable surface TaO_2_ and Ta_2_O_5_. Furthermore, MQDs are spontaneously uptaken into antigen‐presenting endothelial cells and alter surface receptor expression to reduce their activation of allogeneic T‐lymphocytes. Finally, when applied in vivo, Ta_4_C_3_T*_x_* MQDs ameliorate the cellular and structural changes of early allograft vasculopathy. These findings highlight the robust potential of tailored Ta_4_C_3_T*_x_* MQDs for future applications in medicine.

## Graphical abstract

The development and application of new immunoengineered tantalum‐carbide (Ta_4_C_3_T*_x_*) MXene quantum dots (MQDs) for the treatment of transplant vasculopathy are reported. Using rational design and novel synthesis strategies, these MQDs possess intrinsic anti‐inflammatory properties. These findings highlight the robust potential of smart Ta_4_C_3_T*_x_* MQDs for future applications in biomedical engineering and nanomedicine.

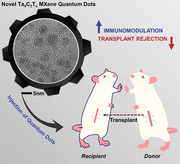

## 1 Introduction

Low‐dimensional carbon‐based nanomaterials are unquestionably the “wonder materials” of today. Since the discovery of graphene in 2004, graphene and its derivatives have been studied extensively in electronic circuits, energy storage, light processing, chemical processing, and biomedical applications.^[^ ^1^, ^2^, ^3^, ^4^, ^5^, ^6^ ^]^ More recently, 0D graphene and MXene quantum dots (MQDs) have been found to possess broad immunomodulatory activity through interactions with a variety of immunologically active cells.^[^ ^7^, ^8^, ^9^, ^10^, ^11^, ^12^ ^]^ In particular, newer MQDs have potential to offer improved dispersibility, tunability, and biocompatibility over traditional graphene materials while maintaining immunomodulatory bioactivity.^[^ ^13^, ^14^, ^15^ ^]^ However, the field remains in a relative infancy and the detailed mechanisms of action of these materials have remained elusive so far.^[^ ^16^ ^]^ Furthermore, currently available evidence is largely based on in vitro studies and MXene materials have not yet been explored in vivo in a clinically relevant inflammatory disease model.

Recently, we reported the biocompatibility and anti‐inflammatory effects of titanium carbide (Ti_3_C_2_T*_x_*) MQDs in low concentrations.^[^ ^11^ ^]^ Notably, these MQDs effectively suppressed proinflammatory T_H_1 polarization of naïve CD4^+^ T‐lymphocytes under synthetic in vitro conditions. These revelations have sparked significant interest in immunoengineering Ti_3_C_2_T*_x_* MXenes for clinical applications.^[^ ^16^ ^]^ In particular, MXene‐based approaches are being developed to treat refractory inflammatory conditions and suppress rejection of transplanted tissue constructs.^[^ ^11^, ^16^ ^]^ However, the long‐term bioinertness of titanium‐based materials has since been called into question.^[^ ^17^, ^18^ ^]^ In fact, several reports on the cytotoxicity of Ti_3_C_2_T*_x_* MXene at medium‐to‐high concentrations raised significant concern on the eventual clinical translatability of these materials.^[^ ^19^, ^20^ ^]^ Future application of this technology therefore hinges on addressing this fundamental limitation.

In response to this challenge, other MXene compositions, such as niobium carbide (Nb_2_C), have been developed with reduced cytotoxic potential.^[^ ^21^, ^22^, ^23^ ^]^ However, niobium has not been commonly used in biomedical applications and their long‐term safety remains poorly understood.^[^ ^24^ ^]^ On the other hand, tantalum‐based biomaterials are well studied and have been previously shown to possess improved corrosion resistance, biocompatibility, and bioactivity over those derived from titanium.^[^ ^25^, ^26^, ^27^ ^]^ In particular, tantalum oxides have been shown to be more stable and inert than their titanium‐based counterparts, which contributes to the excellent biological safety profile of tantalum‐based materials.^[^ ^28^, ^29^, ^30^, ^31^, ^32^, ^33^, ^34^ ^]^ These finding have been corroborated by both in vitro and in vivo experiments showing the safety of high dose tantalum carbide (Ta_4_C_3_T*_x_*) MXene nanosheets (MNSs).^[^ ^35^, ^36^ ^]^ However, the successful synthesis of highly desirable Ta_4_C_3_T*_x_* MQDs has not been reported yet. MQDs are uniquely suitable for biomedical and immunoengineering applications due to their improved aqueous stability and subcellular‐level interactions.^[^ ^37^ ^]^ Development of Ta_4_C_3_T*_x_* MQDs is therefore urgently needed to keep pace with this rapidly evolving field.

Herein, we present the design, fabrication, characterization, and application of immunoengineered tantalum carbide (Ta_4_C_3_T*_x_*) MXene quantum dots for in vitro and in vivo immunomodulatory applications. These Ta_4_C_3_T*_x_* MQDs were rationally designed for biomedical applications through a tailored etching, exfoliation, and hydrothermal process. As‐synthesized MQDs exhibited high concentrations of MXene surface functional groups as well as the surface tantalum oxides (TaO_2_ and Ta_2_O_5_), which contributed to its excellent biocompatibility with human cells. In particular, high concentrations of Ta_4_C_3_T*_x_* MQDs did not induce oxidative stress and cytotoxicity in cultured human endothelial cells (ECs). Furthermore, these MQDs were spontaneously internalized into ECs and mechanistically contributed to reducing the immunogenicity of these cells through regulation of T‐cell activation. Finally, when applied in an in vivo model of organ transplant rejection, intravenous administration of Ta_4_C_3_T*_x_* MQDs reduced both immune cell infiltration and structural degeneration within transplanted tissues. Taken together, this study highlights the future potential of tailored Ta_4_C_3_T*_x_* MQDs in immunoengineering and other biomedical applications.

## 2 Results and Discussion

### 2.1 Rationale, Design, and Synthesis of Ta_4_C_3_T*_x_* MQDs

The engineering of biomedically relevant nanomaterials requires strict control of their chemical composition, structure, and properties.^[^ ^13^ ^]^ The deliberate choice of tantalum‐based MXene in this study arises from considerations of both biocompatibility and bioactivity. Despite ample evidence on the biomedical efficacy of Ti_3_C_2_T*_x_* MXene nanosheets and quantum dots, increasing concerns on their potential cytotoxicity, albeit at higher doses, cannot be ignored. Large quantities of exposed titanium oxides (TiO_2_ and Ti_2_O_3_) can form on the surface of Ti_3_C_2_T*_x_* MQDs during the hydrothermal process or upon aqueous dispersion.^[^ ^38^, ^39^, ^40^ ^]^ Additionally, Ti_3_C_2_T*_x_* MXene products can spontaneously oxidize under ambient storage conditions to form transition metal oxide particles.^[^ ^41^ ^]^ The presence of titanium oxides is particularly concerning in designing materials for biomedical applications, as it can catalyze the production of reactive oxygen species (ROS) and generate oxidative stress to nearby cells and tissues.^[^ ^42^, ^43^, ^44^ ^]^ This surge in ROS also induces the release of proinflammatory cytokines from resident tissue macrophages, which hinders the functionality of immunomodulatory materials.

**Figure 1:**
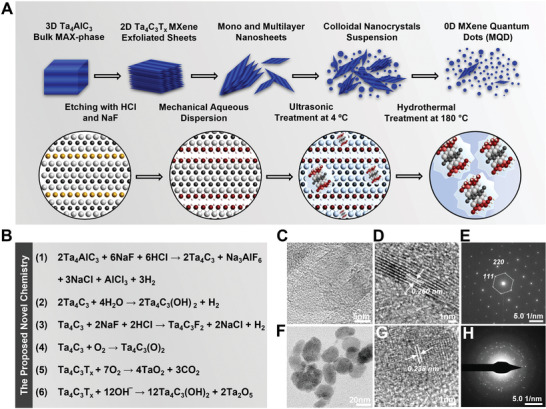
Synthesis schematic model, stoichiometry, and materials characterization. A) Step‐by‐step schematic on the conversation of Ta_4_AlC_3_ MAX phase bulk to 0D Ta_4_C_3_T*_x_* MQDs using a facile protocol. Briefly, Ta_4_AlC_3_ MAX phase powder was etched using an HCl/NaF etchant to remove Al layers and synthesize Ta_4_C_3_T*_x_* MXene nanosheets. This wet etching was performed continuously during the synthesis process for 48 h. Simultaneously, heating at 60 °C enhanced the exfoliation and functionalization of Ta_4_AlC_3_ to form accordion‐like 2D MXene. The exfoliated Ta_4_C_3_T*_x_* nanosheets were further treated by sonication and mechanical vibration to obtain multi‐, oligo‐, and monolayer flakes, which were subsequently treated using a hydrothermal process to form Ta_4_C_3_T*_x_* MQDs with concentrated functional groups as well as stable surface tantalum oxides. B) Proposed reaction chemistry for the synthesis of Ta_4_C_3_T*_x_* MQDs, including surface modification with tantalum oxides (TaO_2_ and Ta_2_O_5_). C–H) Morphology and microstructural characterization of the Ta_4_C_3_T*_x_* MNSs and MQDs. C,D) High‐resolution TEM (HRTEM) images of multilayer Ta_4_C_3_T*_x_* nanosheets revealed well‐defined and exfoliated crystals with lattice *d*‐spacing of ≈0.260 nm. E) Furthermore, the corresponding SAED pattern of the nanosheets depicted a uniform hexagonal crystallization pattern typical for MXene materials. F) HRTEM images of Ta_4_C_3_T*_x_* MQDs displayed proper synthesis of particles with high surface functionalization after hydrothermal treatment at 180 °C. G) As shown, the average diameter of a single Ta_4_C_3_T*_x_* particle is ≈3.5 nm, which is ideal for targeted subcellular applications. H) The lattice *d*‐spacing of MQDs was found to be ≈0.338 nm. Collectively, these data support the successful design and production of the new Ta_4_C_3_T*_x_* MQDs.

Therefore, in the current study, Ta_4_C_3_T*_x_* MQDs were specifically designed with biomedical applications in mind and synthesized using a facile methodology to accommodate these design requirements. The Ta_4_AlC_3_ MAX phase was chemically etched and exfoliated to form accordion‐like Ta_4_C_3_T*_x_* MNSs using hydrochloric acid/sodium fluoride (HCl/NaF) as etchant. The resultant MXene products were subsequently dispersed in pure distilled water and further treated by bath sonication to obtain multi‐, oligo‐, and single‐layered Ta_4_C_3_T*_x_* MXene nanocrystals. Finally, the obtained aqueous colloidal suspension underwent hydrothermal treatment at 180 °C for 12 h to obtain 0D Ta_4_C_3_T*_x_* MQDs. A step‐by‐step schematic of the production of Ta_4_C_3_T*_x_* MQDs is presented in **Figure** **1**A.

Despite milder nature of the HCl/NaF etchant over hydrofluoric acid (HF), effectiveness of this etching process has already been demonstrated for niobium and vanadium carbide (V_2_C) MXenes, which carry similar formation energies to Ta_4_C_3_T*_x_* MXene.^[^ ^45^, ^46^, ^47^ ^]^ Furthermore, there are several distinct advantages to this approach. First, the fluoride salt etchant is expected to produce fewer surface defects than HF treatment, thereby reducing opportunities for oxidative degradation and increasing the stability and shelf‐life of the end product.^[^ ^41^, ^48^ ^]^ Second, this etching process facilitates intercalation of cations and water between the MXene layers, thereby weakening interlayer interactions.^[^ ^48^ ^]^ This results in expansion of the interlayers spacing in MXene nanosheets and facilitates the subsequent delamination process. Last, this approach reduces the manufacturing challenges associated with use of concentrated HF while maintaining the strict tunability of the MXene end products. In this study, NaF was specifically chosen over the conventional LiF due to cytotoxic concerns associated with lithium moieties in the structure of MXenes.^[^ ^44^ ^]^

Furthermore, the Ta_4_C_3_T*_x_* MXene nanosheets were treated by ultrasonication and subsequent homogenization to enhance its specific surface area and aqueous colloidal dispersibility. In particular, mechanical vibration and/or sonication treatment increases the degree of cationic intercalation and further increases interlayer spacing. As a result, the obtained colloidal solutions contained well dispersed and electrostatically stabilized MXene nanosheets. Furthermore, colloidal suspensions of MXene flakes produced from this process are less likely to clump or aggregate, thereby increasing its accessibility for further functionalization.^[^ ^49^ ^]^ This protocol, therefore, offers potential for the industrial development of bioactive and clinically translatable Ta_4_C_3_T*_x_* MQDs.

### 2.2 Proposed Reaction Chemistry for Synthesis of Ta_4_C_3_T*_x_* MQDs

The proposed chemical reactions for the synthesis of Ta_4_C_3_T*_x_* MQDs in the above described fabrication process are presented in Figure 1B. Exfoliation of the Ta_4_AlC_3_ MAX phase was achieved using a two‐step approach. First, the MAX phase powder was chlorinated by treatment with 12 m HCl at 60 °C to significantly remove the surface Al layer through formation of aluminum chloride (AlCl_3_). Additionally, the presence of NaF in the etching solution completed the exfoliation process through the formation of sodium hexafluoroaluminate (Na_3_AlF_6_), further removing any remaining Al traces. This led to successful production of multilayered Ta_4_C_3_T*_x_* MXene nanosheets (Figure 1B, Equation (1)). Moreover, the presence NaF during the agitation process resulted in further surface functionalization of the MXene layers with abundant —OH groups. Furthermore, the proposed reaction chemistry also supports effective fluorination of the end product with rich —F surface terminals. Together, these mechanisms of reactions enabled facile exfoliation of Ta_4_AlC_3_ MAX phase powder and efficient synthesis of 2D Ta_4_C_3_T*_x_* MXene nanosheets (Figure 1B, Equations (1)–(3)).

The chemical reactions that occurred during functionalization process of the colloidal dispersions arose after partial oxidation of Ta_4_C_3_T*_x_* MNSs in aqueous media (Figure 1B, Equation (4)). The hydrothermal process led to the formation of tantalum oxide (both TaO_2_ and Ta_2_O_5_) layers on the surface of Ta_4_C_3_T*_x_* MQDs, presumably through a secondary crystal nucleation mechanism (Figure 1B, Equations (5) and (6)). It is important to note that these chemical reactions also facilitate the formation of additional —OH and =O groups on the surface of MQDs after hydrothermal treatment. Additionally, chemical interactions of existing Cl‐, F‐, and Na‐based compounds with the surface of Ta_4_C_3_T*_x_* are expected to occur during the synthesis process as well. Therefore, different stable surface functional groups can be readily identified on the surface of MQDs, supporting its chemical stability and bioactivity.

### 2.3 Microstructural Characterization of Ta_4_C_3_T*_x_* MQDs

Successful synthesis of Ta_4_C_3_T*_x_* MQDs using our innovative synthetic process was confirmed using transmission electron microscopy (TEM), fast Fourier transform (FFT) analysis, selected area diffraction (SAED) analysis, and energy‐dispersive X‐ray spectroscopy (EDS). Scanning electron microscopy (SEM) images of the Ta_4_AlC_3_ MAX phase and its corresponding EDS analysis showed bulky morphology with an atomic percentage of 19.77% for Al in the composition (Figure S1, Supporting Information). TEM images of Ta_4_C_3_T*_x_* nanosheets after treatment with HCl/NaF demonstrated stacked basal planes of the MXene layers (Figure 1C). Additionally, high‐resolution TEM (HRTEM) and SAED images of exfoliated Ta_4_C_3_T*_x_* MXene flakes confirmed a symmetric crystalline structure consisting of layers with *d*‐spacing of ≈0.260 nm, which can be assigned to (111) plane (Figure 1D,E). Notably, the obtained hexagonal lattice *d*‐spacing of Ta_4_C_3_T*_x_* MXene nanosheets agreed well with previous reports, confirming that the planar structure of sheets remained stable and unperturbed during the synthesis process.^[^ ^50^ ^]^ Furthermore, EDS elemental and mapping analysis of this sample clearly depicted significant removal of Al from the structure of MAX phase (Figure S2, Supporting Information).

The subsequent mechanical processing and hydrothermal treatment at 180 °C resulted in successful formation of surface functionalized Ta_4_C_3_T*_x_* MQDs (Figure 1F). HRTEM images of the acquired MQDs revealed well‐defined quantum structure with a particle size of less than 5 nm in diameter (Figure 1G; Figure S3A,B, Supporting Information). Furthermore, Ta_4_C_3_T*_x_* MQDs exhibited a highly crystalline diffraction pattern with multiple differently oriented planes. In particular, the corresponding SAED/FFT patterns displayed a crystalline structure of MQDs with an atomic *d*‐spacing of ≈0.338 nm (inner plane: ≈0.238 nm; Figure 1H; Figure S3C, Supporting Information). Notably, amorphous rings were seen in the SAED analysis of Ta_4_C_3_T*_x_* MQDs and can be attributed to remaining noncrystalline carbon‐based particles.^[^ ^38^ ^]^ Interestingly, these properties were unchanged when the material was evaluated more than 1 year after the synthesis, supporting high microstructural stability of the colloidal Ta_4_C_3_T*_x_* MQDs (Figure S3D–F, Supporting Information). Finally, EDS elemental analysis of Ta_4_C_3_T*_x_* MQDs retained the low atomic percentage of Al at less than 1%, which is consistent with what was seen in the MNSs (Figure S4, Supporting Information). Together, these observations provide robust evidence that the HF‐free etching and subsequent hydrothermal treatment employed in this study has successfully fabricated highly stable crystalline Ta_4_C_3_T*_x_* MQDs.

**Figure 2:**
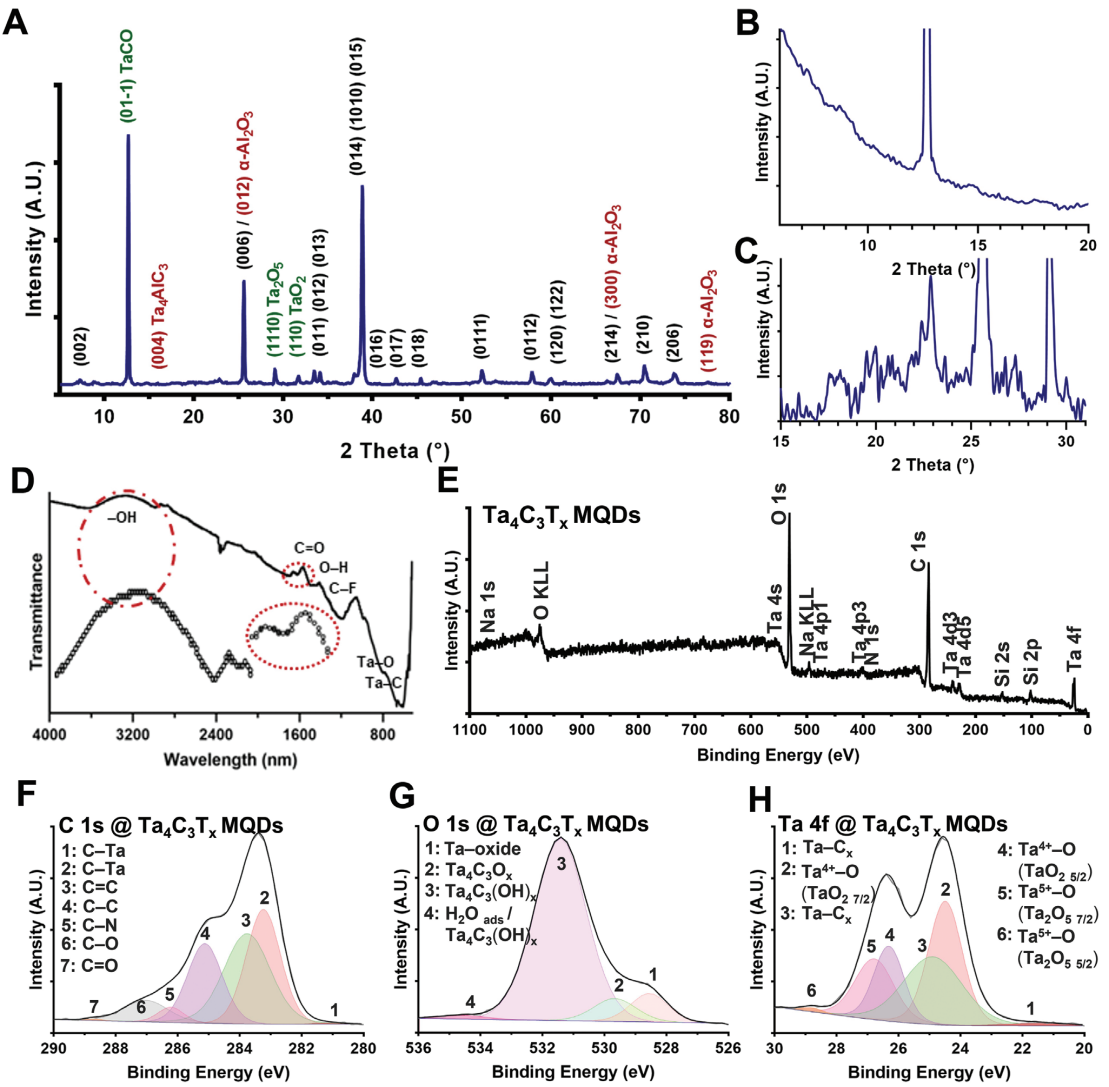
Characterization of the structure, functional groups, and chemical composition of as‐synthesized Ta_4_C_3_T*_x_* MQDs. A–C) XRD phase characterization of Ta_4_C_3_T*_x_* MQDs was performed at 5° to 80° 2θ. Our XRD data displayed the main characteristic (002) peak of the MXene materials at ≈7° 2θ in the Ta_4_C_3_T*_x_* MQDs. High‐resolution XRD analysis of the MQDs depicted a remarkable downshift of the Al‐containing peaks, which was a robust confirmation for the synthesis of Ta_4_C_3_T*_x_* MXenes. The peaks were mainly matched according to the standard reference codes for Ta carbide, Ta‐oxide carbide, and Ta oxide phases (96‐210‐3218, ICSD156383, and α‐alumina). D) FTIR analysis of the MQDs. Our FTIR data showed clear formation of additional functional groups on the surface of MQDs. The FTIR spectra displayed a broad peak at ≈3500 cm^−1^, which may be attributable to expansion of lattice parameters in the MQDs. E) The wide‐scan XPS spectrum of these MQDs revealed that the main Al 2p and Al 2s peaks at the binding energies of 64–80 and 115–125 eV were significantly extracted from the material composition during synthesis process. Our XPS survey also showed well‐defined MXene characteristics (Ta 4f, Ta 4p, C 1s, O 1s, Cl 2p, Na 1s, and F 1s) with a high degree of surface functionalization in comparison with its MAX phase structure (Figure S5, Supporting Information). F–H) Furthermore, the narrow spectra of Ta 4f, C 1s, and O 1s confirmed that Ta_4_C_3_T*_x_* MQDs were successfully synthesized. A detailed peaks identification and elemental composition of the XPS fitting analysis of MQDs is presented in Table S1 in the Supporting Information. Overall, the high‐resolution narrow spectra of Ta_4_C_3_T*_x_* MQDs are supported by our XRD and other characterizations.

To further characterize the structural transformation of MAX phase to Ta_4_C_3_T*_x_* MQDs, X‐ray diffraction (XRD) analysis was performed. Our data demonstrated that the main characteristic (002) peak of MXene clearly emerged at ≈7° 2θ in the Ta_4_C_3_T*_x_* MQDs sample (**Figure** **2**A,B). Simultaneously, the Ta_4_AlC_3_ peaks were significantly downshifted after the exfoliation and hydrothermal process. In particular, one of the main MAX phase peaks at ≈16° 2θ was completely removed from the XRD spectra of Ta_4_C_3_T*_x_* MQDs (Figure S5, Supporting Information). Additionally, a minor amorphous curve between 10° and 30° 2θ was identified in the XRD pattern of Ta_4_C_3_T*_x_* MQDs (Figure 2B,C). As described in the previous section, this change reflects the remaining carbon dots formed during the hydrothermal process. Furthermore, a contamination peak of tantalum carbide (Ta_2_C) in the XRD spectrum of the MAX phase at ≈50° 2θ disappeared completely in the XRD spectrum of Ta_4_C_3_T*_x_* MQDs, reflecting the efficiency of synthesis and purity of the end product.

A new dominant peak was seen at ≈12° 2θ in the Ta_4_C_3_T*_x_* MQDs, corresponding to the addition of a crystalline tantalum carbide‐based oxide composite in the structure of MQDs. Identification of alpha‐alumina (α‐Al_2_O_3_) peaks in the XRD pattern of MQDs provided further evidence for effective removal of Al and the conversion of its remaining traces to oxide form. Additionally, there were two new precise peaks, which emerged in the XRD spectra of the MQDs, corresponding to TaO_2_ (110) and Ta_2_O_5_ (010). The detection of transition metal oxide formation during hydrothermal process reflects both theoretical and experimental evidence available in the literature.^[^ ^38^ ^]^ Furthermore, the XRD pattern of the Al‐etched Ta_4_C_3_T*_x_* revealed enlarged lattice spacing in the atomic structure of MQDs (Figure 2B). This expansion is largely attributed to surface functionalization during the synthesis process. Thus, these characterizations demonstrated successful production of surface modified Ta_4_C_3_T*_x_* MQDs.

Next, the surface functional groups of Ta_4_C_3_T*_x_* MQDs were evaluated using Fourier‐transform infrared spectroscopy (FTIR). The FTIR spectrum of Ta_4_C_3_T*_x_* MQDs identified characteristic Ta—C, Ta—O, and Ta—F bonds of Ta_4_C_3_T*_x_* MXenes (Figure 2D). Additionally, FTIR assessment revealed the vibrations of key surface functional groups, including —OH, C—F, C=O, Ta—C, Ta—O, and Ta—F, available in the structure of Ta_4_C_3_T*_x_* MQDs. The FTIR stretching of these bonds was detected at the wavelengths of ≈500 to 3500 nm. The presence of a C–F peak in the spectrum of Ta_4_C_3_T*_x_* MQDs at ≈1200 cm^−1^ suggested efficient fluorination of particles during synthesis process. Additionally, a weak vibration was identified at ≈3100 cm^−1^ that may suggest the presence of amine functional group (—NH_2_).^[^ ^11^ ^]^ This characterization confirmed the presence of quantities of function groups on the surface of Ta_4_C_3_T*_x_* MQDs and indicated the successful fabrication of functionalized particles.

Last, X‐ray photoelectron spectroscopy (XPS) was used to characterize the surface‐bonding structures of Ta_4_C_3_T*_x_* MQDs. Wide‐scan survey comparison of Ta_4_AlC_3_ MAX phase and its derived product revealed the generation of high quality MQDs during the synthesis process (Figure 2E; Figure S6A, Supporting Information). In particular, the Al 2p narrow scan XPS spectrum of Ta_4_C_3_T*_x_* MQDs confirmed the efficacy of etching process in removing Al layers from the structure of the MAX phase (Figure S6B, Supporting Information). Additionally, the high‐resolution C 1s spectrum of Ta_4_C_3_T*_x_* MQDs contained the combination of Ta—C*_x_*, C—C, C—N, C=C, C—O, and C=O peaks fitted at binding energies between 280 and 290 eV (Figure 2F). Additionally, the O 1s spectrum identified oxygen‐containing peaks located between 526 and 536 eV, signifying a high level of oxygen‐containing functional groups on the surface of these MQDs (Figure 2G).

The Ta 4f narrow scan of Ta_4_C_3_T*_x_* MQDs displayed prominent 4f 5/2 and 4f 7/2 peaks of tantalum and tantalum oxide at the binding energies of 20 to 30 eV (Figure 2H). In particular, formation of lateral species of Ta^4+^ and Ta^5+^ as main Ta‐oxide structures (TaO_2_ and Ta_2_O_5_) was detected at binding energies of ≈24 to 25 eV. Furthermore, in agreement with the proposed chemical reactions, XPS high‐resolution spectra of Cl 2p, Na 1s, and F 1s demonstrated the formation of additional surface functions on MQDs by the HCl/NaF etchant (Figure S6C–E, Supporting Information). In particular, the Cl 2p spectrum exhibited a combination of metal‐chloride peaks (3/2 and 1/2) at binding energies of ≈197 to 200 eV and nonmetal Cl at ≈201 eV. Moreover, the XPS spectra of Na 1s depicted a dominant peak at ≈1071 eV assigned to Na^+^ ions. This region contains two peaks at the binding energies of ≈1067 and 1069 eV corresponding to the interaction of Na^+^ with Ta‐oxide and Ta—C, respectively. Additionally, as discussed in the previous section, the rationally designed synthesis protocol enabled a mechanism to fluorinate the surface of Ta_4_C_3_T*_x_* MQDs, as confirmed by the high‐resolution F 1s spectrum. Of note, two peaks were fitted at binding energies of ≈684 and 687 eV, attributed to Ta—F (atomic percentage: 71.82%) and C—F (atomic percentage: 28.18%), respectively. These measurements served as another confirmation to successful surface modification of Ta_4_C_3_T*_x_* MQDs during the synthesis process. Detailed quantification of the identified Ta–O, Ta–C*_x_*, C–O, C=O, C–N, C=C, Ta_4_C_3_O*_x_*, Ta_4_C_3_(OH)*_x_*, Ta–F, C–F, Ta–Cl, and Na^+^ groups of MQDs is presented in Table S1 in the Supporting Information. Overall, the XPS analysis of as‐synthesized Ta_4_C_3_T*_x_* MQDs agreed very well with our XRD and FTIR characterizations and confirmed that the employed synthesis procedure reported in the current study was highly efficient and suitable for targeted applications.

### 2.4 Thermal, Optical, and Surface Properties of Ta_4_C_3_T*_x_* MQDs

**Figure 3:**
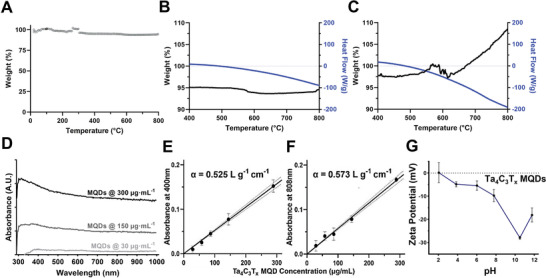
Thermophysical and optical absorption properties of Ta_4_C_3_T*_x_* MQDs. A–C) TGA/DSC analysis of Ta_4_C_3_T*_x_* MQDs in argon and atmospheric conditions. A) Under argon gas, TGA analysis of Ta_4_C_3_T*_x_* MQDs demonstrated no significant effects on the surface terminations or decomposition of material after annealing of Ta_4_C_3_T*_x_* MQDs at up to 800 °C. B) Furthermore, our data showed that there was no significant mass loss after 350 °C, and the char residue was higher than 90% in this temperature range. C) However, under normal atmospheric conditions, the TGA curve of Ta_4_C_3_T*_x_* MQDs showed a steady increase in mass percentage due to the oxidation process after ≈600 °C. D) The UV–visible spectrum of the Ta_4_C_3_T*_x_* MQDs demonstrated a strong dose‐dependent absorption in the area of ≈300 nm, corresponding to the lateral carbon structure, and an additional broad absorption peak at around 900 nm. E,F) The calculated α for the novel Ta_4_C_3_T*_x_* MQDs was measured to be 0.525 and 0.573 L g^−1^ cm^−1^ at 404 and 808 nm, respectively. G) The zeta potential of Ta_4_C_3_T*_x_* MQDs was largely negative across the pH range of 2 to 12 (around 0 to −30 mV). A slight increase in surface charge of the MQDs was noted at pH higher than 10, likely due to property changes in the functional groups of particles under strong alkaline conditions.

The surface properties of MXene nanostructures are defined by their synthesis conditions. In this regard, effective fabrication methods must be applied to obtain MXene materials with the desired surface terminations and long‐term stability. Previously, thermogravimetric analysis (TGA) has been used to characterize the temperature‐dependent desorption of surface terminations of MXene materials.^[^ ^51^ ^]^ This method can effectively quantify the thermal stability of surface functional groups and terminations after annealing. In the current study, the stability of Ta_4_C_3_T*_x_* MQDs was evaluated using TGA. Under vacuum condition, the —OH functional group is the first species to desorb from the surface of heated MXenes, starting at temperatures above 300 °C.^[^ ^52^ ^]^ Notably, the signal of —OH desorption from the MXene samples can be significantly masked by the release of deprotonated H_2_O during TGA measurement. However, defunctionalization of surface —OH groups in the structure of MXenes subsequently lead to partial electron transformation of this group into more stable oxygen‐containing functional species. Our TGA curve of Ta_4_C_3_T*_x_* MQDs depicted a slight mass‐loss between ≈150 and 300 °C, and its char residue was 10% at around 600 °C (**Figure** **3**A). However, our TGA data showed almost no mass loss after 350 °C, and in this temperature range, its char residue was as higher than 91% (Figure 3B).

Interestingly, our TGA data demonstrated that annealing Ta_4_C_3_T*_x_* MQDs up to 800 °C had no significant effects on its surface termination and did not result in significant decomposition of material. A minor mass gain (≈1%) was observed at annealing temperature above 800 °C and can most likely be attributed to oxidation of impurities or slight decomposition of the material. In contrast, TGA curve of Ta_4_C_3_T*_x_* MQDs generated under normal atmospheric conditions showed a continuous increase in its mass percentage due to oxidation process, starting at ≈600 °C (Figure 3C). As apparent in our results, the heat flow data of differential scanning calorimetry (DSC) curves are in good agreement with the obtained TGA in both argon and atmospheric conditions (Figure 3B,C). Together, this data supports that the employed HF‐free protocol in the current study was able to successfully synthesize highly stable Ta_4_C_3_T*_x_* MQDs.

Furthermore, the MXene materials are relatively transparent in visible lights and are known to possess excellent optical properties.^[^ ^53^ ^]^ These specific properties precisely depend on the tailored intercalation, interlayer spacing, and surface architecture of individual MXene materials. Therefore, surface modification and functionalization during the synthesis of MXene structures can be used to achieve desired absorption and optical properties. To this regard, we assessed the UV–visible absorption spectra of an aqueous colloidal dispersion of Ta_4_C_3_T*_x_* MQDs. In particular, MQDs at different concentrations of ≈30 to 300 µg mL^−1^ were examined to characterize their optical properties. The UV–visible spectrum of Ta_4_C_3_T*_x_* MQDs at 230 to 990 nm demonstrated a clear dose‐dependent absorption profile for the dispersed particles (Figure 3D–F; Figure S7A, Supporting Information). Strong absorption was noted in the area of ≈300 nm, corresponding to the lateral carbon structure of Ta_4_C_3_T*_x_* MQDs. In fact, due to the colloidal nature of Ta_4_C_3_T*_x_* MQDs, a linear correlation between absorption and concentration of particles could be observed for the MQDs, which can be described using the Beer–Lambert law (Equations (S1) and (S2), Supporting Information). Based on the measured standard curves, the α value was calculated to be 0.525 and 0.573 L g^−1^ cm^−1^ at 404 and 808 nm, respectively, which can be used as a robust parameter for future studies using Ta_4_C_3_T*_x_* MQDs. Subsequently, the long‐term colloidal stability of aqueous MQDs dispersion was further confirmed six months after the initial synthesis and characterization (Figure S7B, Supporting Information). As shown in these optical micrographs, the developed environment‐friendly protocol in the current study resulted in the fabrication of stable surface‐modified and uniform MQD suspensions without significant stacking and agglomeration of the particles at the test concentrations of 250 µg mL^−1^.

The surface charge of synthetic nanomaterials also has a significant effect on their bioactive properties.^[^ ^54^ ^]^ In the next experiment, the surface charge behavior of the Ta_4_C_3_T*_x_* MQDs was assessed at a concentration of 75 µg mL^−1^ and different pH values. The zeta potential (ζ) data in the current study suggested that as‐synthesized Ta_4_C_3_T*_x_* MQDs have a surface charge between −5 and −10 mV at pH 7 (Figure 3G). However, the MQDs exhibit pH‐dependent change in the surface charge, with the point of zero charge (PZC) at a pH of ≈2 and progressively more negative surface charge at higher pHs. This observation can be explained by the abundance of surface carboxyl groups, which demonstrate pH‐dependent ionization, and is consistent with the previously reported analysis of other MXene counterparts.^[^ ^55^ ^]^ Notably, the zeta measurements in the current study revealed a slight increase (around 10%) in the surface charge of the MQDs from pH 10 to 12, which may be attributed to changes in the structure of particles or surface functional groups under strong alkaline conditions. Nevertheless, the negative surface charge of these Ta_4_C_3_T*_x_* MQDs likely contributes to the bioactivity of these quantum dots through facilitation of material–cell interactions. Furthermore, we assessed the electrical conductivity of aqueous, colloidal suspension of Ta_4_C_3_T*_x_* MQDs. At a concentration of 250 µg mL^−1^, the MQDs showed a remarkably high electrical conductivity of 10 543 ± 77 µS cm^−1^ (Figure S8, Supporting Information). Taking all these accounts together, the data strongly supports the successful development of a new nontoxic Ta_4_C_3_T*_x_* MQDs functional material with excellent microstructure and surface properties for targeted biomedical and other potential applications, including theranostic, cancer therapy, regenerative nanomedicine, electronic, and water filtration.

### 2.5 Biocompatibility of Ta_4_C_3_T*_x_* MQDs

The biocompatibility of as‐synthesized Ta_4_C_3_T*_x_* MQDs was assessed in vitro using cocultures with human umbilical vein endothelial cells (HUVECs). The ECs form the lining of blood vessels and serve as the first point of contact between the body and intravenously delivered nanomaterials. These cells also play important roles in the regulation of inflammation, coagulation, and nutrient delivery to different tissues. Endothelial toxicity can therefore significantly limit the future biomedical applications of nanomaterials.^[^ ^23^ ^]^

**Figure 4:**
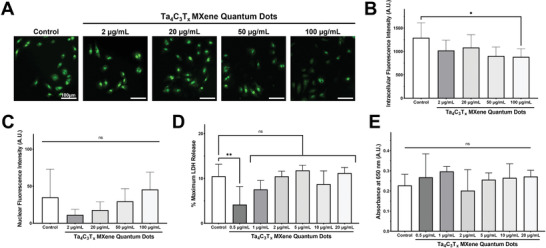
Evaluation of the reactive oxygen species (ROS) generation and biocompatibility of Ta_4_C_3_T*_x_* MQDs. A) Total cellular ROS was evaluated in HUVECs using a green fluorescence probe. B) Culture with Ta_4_C_3_T*_x_* MQDs at concentrations of 2 to 100 µg mL^−1^ did not increase total cellular ROS when compared to the control. C) Additionally, caspase‐3/7 activation was assessed using a green fluorescent probe, and Ta_4_C_3_T*_x_* MQDs at concentrations of 2 to 100 µg mL^−1^ did not increase apoptotic activation when compared to the control. D) Biocompatibility at 3 days was assessed at different MQD concentrations (0.5 to 20 µg mL^−1^) using the LDH release assay. No significant increases were observed in maximum LDH release between MQD‐treated groups and the control. E) Biocompatibility at 7 days was assessed at different MQD concentrations (0.5 to 20 µg mL^−1^) using the WST‐1 Cell Proliferation assay. No significant differences were observed in cellular proliferation between the MQD‐treated groups and the control.

Furthermore, previous studies have reported that other forms of MXenes, such as Ti_3_C_2_T*_x_* MXenes, can increase cellular ROS levels, create oxidative stress to nearby cells, and cause cellular damage.^[^ ^19^, ^39^, ^40^, ^41^ ^]^ This increase in ROS also induces the release of proinflammatory cytokines from resident tissue macrophages and interferes with anti‐inflammatory and immunomodulatory properties of implanted biomaterials in the body.^[^ ^56^ ^]^ Therefore, in the current study, we first assessed if the presence of Ta_4_C_3_T*_x_* MQDs causes any ROS generation in cells. The HUVECs were cultured with or without different doses of Ta_4_C_3_T*_x_* MQDs (2 to 100 µg mL^−1^ in phosphate‐buffered saline (PBS)) for 24 h. The intracellular ROS levels were assessed using the CellROX green fluorescent dye. It is evident from our data that Ta_4_C_3_T*_x_* MQDs did not increase intracellular ROS levels across the concentration range used in this study (**Figure** **4**A,B). In fact, the highest concentration of MQDs (100 µg mL^−1^) appeared to attenuate the oxidative stress compared to vehicle control group (Figure 4A,B). These data highlight the unique advantages afforded by the structural composition of Ta‐based MXenes over their titanium counterparts.

Next, the biocompatibility of Ta_4_C_3_T*_x_* MQDs with HUVECs was investigated using the CellEvent fluorescence‐based apoptosis detection kit that detects the activities of caspase‐3 and caspase‐7. These two caspases are the primary executioners of programmed cell death in cells subject to insurmountable stressful conditions. The activation of both caspase‐3 and caspase‐7 are reported to be associated with cellular apoptosis and cell death. In the current study, HUVECs were subjected to nutrient deprivation and subsequently cultured with Ta_4_C_3_T*_x_* MQDs for 24 h. As shown in Figure 4C, Ta_4_C_3_T*_x_* MQDs up to 100 µg mL^−1^ exhibited no significant activation of caspase‐3 and caspase‐7 when compared with the controls. Therefore, Ta_4_C_3_T*_x_* MQDs are cyto‐compatible and do not cause any cellular damage. Furthermore, among the concentrations used for subsequent studies (0.5 to 20 µg mL^−1^), no significant differences were observed in cellular cytotoxicity and proliferation at any Ta_4_C_3_T*_x_* MQD concentrations for up to 7 days (Figure 4D,E; Figure S9, Supporting Information). These findings further establish the importance of rationally designed and synthesized Ta_4_C_3_T*_x_* MQDs for future biomedical applications.

### 2.6 Immunomodulatory Properties of Ta_4_C_3_T*_x_* MQDs

**Figure 5:**
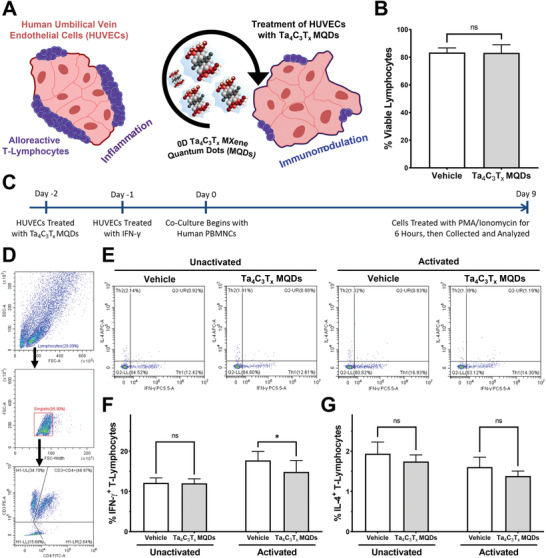
In vitro evaluation of immunomodulatory effects of Ta_4_C_3_T*_x_* MQDs. A) Schematic representation of the in vitro immunomodulatory model. Ta_4_C_3_T*_x_* MQDs interact with HUVECs to reduce inflammatory activation of cocultured lymphocytes. B) Cytotoxicity assessment of Ta_4_C_3_T*_x_* MQDs on human lymphocytes. No significant cytotoxicity was observed when lymphocytes were treated with 2 µg mL^−1^ of MQDs. C) Schematic showing the timeline for in vitro immunomodulation assessment. HUVECs were pretreated with Ta_4_C_3_T*_x_* MQDs, activated with IFN‐γ, and then cocultured with human lymphocytes for 9 days. D) Flow cytometric gating strategy for analysis of immunomodulation. Single lymphocytes were gated on the CD3^+^CD4^+^ gate. E) Intracellular staining for IFN‐γ and IL‐4 were used to identify T_H_1 and T_H_2 T‐helper cells, respectively. F) Treatment with 2 µg mL^−1^ of Ta_4_C_3_T*_x_* MQDs reduced the percentage of T_H_1 cells when human lymphocytes were cocultured with activated HUVECs. G) No significant differences were observed in the percentages of T_H_2 cells in the coculture experiment.

The immunomodulatory properties of Ta_4_C_3_T*_x_* MQDs were investigated in vitro using cocultures of activated HUVECs and human peripheral blood mononuclear cells (PBMNCs). As the barrier between blood and tissues, ECs play a critical role in the pathophysiology of organ transplant rejection. After allo‐transplantation (donor‐derived), ECs are activated and act as antigen‐presenting cells to the recipient immune system, leading to immune activation, vascular injury, and subsequent rejection of the allograft (donor organ).^[^ ^57^, ^58^ ^]^ In particular, recruitment of proinflammatory type 1 T helper (T_H_1) cells is critical to the development and progression of allograft rejection.^[^ ^59^ ^]^ Thus, in this study, we examined the immunomodulatory effects of Ta_4_C_3_T*_x_* MQDs using activated HUVECs, PBMNCs, and T_H_1 cells as a model for organ transplant rejection (**Figure** **5**A).

First, Ta_4_C_3_T*_x_* MQDs at a concentration of 2 µg mL^−1^ were cultured with antibody‐activated and T_H_1‐directed human PBMNCs in the absence of HUVECs to look for direct immunomodulatory effects. We have previously reported for the first time that titanium carbide (Ti_3_C_2_T*_x_*) MQDs display direct immunomodulatory effects.^[^ ^11^ ^]^ As shown in Figure S10 in the Supporting Information, Ta_4_C_3_T*_x_* MQDs do not appear to exert statistically significant immunomodulatory effects on their own, in the absence of ECs as antigen‐presenting cells, after 1 week of culture. Specifically, no differences were observed in the percentage of interferon‐gamma (IFN‐γ) expressing T‐lymphocytes (Control 74.3%, MQDs 76.3%, ns) or in the proliferation of T‐lymphocytes (Control 36.8‐fold, MQDs 37.3‐fold, ns). Furthermore, these experiments also confirmed the biocompatibility of Ta_4_C_3_T*_x_* MQDs with human lymphocytes, with no obvious differences in T‐cell viability seen after 1 week of culture (Control 83.4%, MQDs 83.2%, ns; Figure 5B). These findings are congruent with previous reports on graphene quantum dots (GQDs), which also possess no direct immunomodulatory effects on lymphocytes and require antigen‐presenting cells to exert their effects.^[^ ^8^ ^]^

To test this hypothesis, HUVECs were treated with Ta_4_C_3_T*_x_* MQDs at 2 µg mL^−1^ for 24 h prior to activation with IFN‐γ at 10 units mL^−1^ for 24 h. As shown in Figure S11 in the Supporting Information, robust activation was achieved at this time point with significant upregulation of human leukocyte antigen class II (HLA‐DRα). These cells were subsequently cocultured with PBMNCs in medium containing Ta_4_C_3_T*_x_* MQDs at 2 µg mL^−1^ and interleukin‐2 (IL‐2) at 5 ng mL^−1^ for 9 days (Figure 5C). As shown in Figure 5D–G, Ta_4_C_3_T*_x_* MQDs exerted distinct immunomodulatory effects on T‐lymphocytes through activated HUVECs. In particular Ta_4_C_3_T*_x_* MQDs significantly reduced the percentage of IFN‐γ^+^ T_H_1 cells among the CD4^+^ T‐lymphocyte population after coculture with activated HUVECs (Vehicle 17.7%, MQDs 14.9%, *p* < 0.05; Figure 5F). These effects were not seen in the unactivated HUVEC group (Vehicle 12.15%, MQDs 12.04%, ns). Interestingly, no significant differences were observed in the proportion of interleukin‐4 (IL‐4) expressing type 2 T helper (T_H_2) cells among cocultures with both activated (Vehicle 1.6%, MQDs 1.4%, ns; Figure 5G) and unactivated (Vehicle 1.9%, MQDs 1.7%, ns) HUVECs. These findings are in line with previous reports, which showed that GQDs interact with antigen‐presenting dendritic cells to reduce the proportion of proinflammatory IFN‐γ^+^ T_H_1 cells after in vitro stimulation.^[^ ^8^ ^]^ However, unlike GQDs, Ta_4_C_3_T*_x_* MQDs do not induce upregulation of T_H_2 T‐lymphocytes. T_H_2 cells are known to exacerbate allergic reactions and contribute toward activation of the humoral immune system.^[^ ^60^, ^61^ ^]^ Thus, these findings strongly support the hypothesis that Ta_4_C_3_T*_x_* MQDs can produce beneficial immunomodulatory effects in clinically relevant models.

### 2.7 Mechanism of Immunomodulation by Ta_4_C_3_T*_x_* MQDs

**Figure 6:**
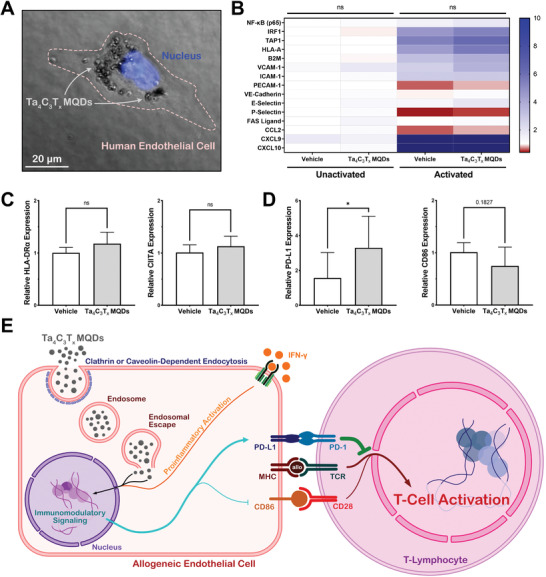
Mechanistic evaluation of the immunomodulatory effects of Ta_4_C_3_T*_x_* MQDs. A) Light microscopy demonstrated that Ta_4_C_3_T*_x_* MQDs were readily internalized into HUVECs after 24 h of culture. B,C) Quantitative PCR analysis was used against genes involved in antigen presentation, cellular adhesion, lymphocyte recruitment, and chemokine signaling. Activation of HUVECs using IFN‐γ resulted in an increase in proinflammatory signaling. No significant differences were observed between cells treated with 20 µg mL^−1^ of Ta_4_C_3_T*_x_* MQDs and the vehicle control. D) Treatment with Ta_4_C_3_T*_x_* MQDs was found to alter the expression of the T‐cell co‐inhibitor PD‐L1 and the T‐cell coactivator CD86 on the surface of activated HUVECs. A significant increase was noted in the endothelial expression of PD‐L1, and a trend toward a decrease of CD86 was observed after treatment with 20 µg mL^−1^ of Ta_4_C_3_T*_x_* MQDs. E) Schematic representation of the immunomodulatory mechanisms of Ta_4_C_3_T*_x_* MQDs. MQDs are internalized into cells through active endocytosis, after which their surface architecture facilitates endosomal escape. They then participate in immunomodulatory signaling to alter the ratio of surface coactivator and co‐inhibitors, which subsequently results in reduced T‐cell activation.

To understand the mechanisms of immunomodulation through Ta_4_C_3_T*_x_* MQDs, the direct interaction of Ta_4_C_3_T*_x_* MQDs with HUVECs were investigated. Interestingly, it was discovered in the current study that Ta_4_C_3_T*_x_* MQDs were rapidly uptaken by endothelial cells and localize near the nucleus of the cell (**Figure** **6**A). The abundance of negatively charged hydroxyl‐, carboxyl‐, chlorine‐, fluorine‐, and amine‐based functional groups on the surface of Ta_4_C_3_T*_x_* MQDs might have facilitated this internalization.^[^ ^62^ ^]^ Furthermore, pH‐dependent changes in the surface charge of Ta_4_C_3_T*_x_* MQDs (Figure 3G) might have facilitated their endosomal escape shortly after internalization.^[^ ^63^ ^]^ As Ta_4_C_3_T*_x_* MQDs become less negatively charged, they can interact with the membrane of the endosomes to escape into the cytoplasm. This ultimately allows them to interact with nuclear and cytoplasmic proteins and participate in subsequent immunomodulatory signaling.

To gain insight into the mechanisms of immunomodulatory signaling induced by Ta_4_C_3_T*_x_* MQDs, a quantitative polymerase chain reaction (qPCR)‐based gene expression analysis of common immunologic pathways was performed in HUVECs (Figure 6B,C). As shown here (Figure 6B,C), Ta_4_C_3_T*_x_* MQDs do not significantly alter expression of genes related to antigen presentation (IRF1, TAP1, HLA‐A, B2M, HLA‐DRα, CIITA), cellular adhesion (PECAM‐1, VE‐Cadherin), lymphocyte recruitment (VCAM‐1, ICAM‐1, E‐Selectin, and P‐Selectin), or chemokine signaling (CCL‐2, CXCL9, CXCL10). Rather, a significant shift in the expression pattern of surface co‐stimulatory and co‐inhibitory molecules in ECs was observed in the current study. As shown in Figure 6D, there was a 3.3‐fold increase in the expression level of the programmed death ligand 1 (PD‐L1) in activated HUVECs treated with Ta_4_C_3_T*_x_* MQDs compared with those treated with the vehicle (*p* < 0.05). Simultaneously, there was a trend toward a 1.3‐fold decrease in the expression level of the CD86 in activated HUVECs treated with Ta_4_C_3_T*_x_* MQDs (*p* = 0.18). Both PD‐L1 and CD86 are reported to be involved in T‐cell activation pathways via antigen presenting cells. PD‐L1 acts as a co‐inhibitor to T‐cell activation while CD86 acts as a coactivator.^[^ ^64^, ^65^ ^]^ Therefore, by altering the relative expression of PD‐L1 and CD86 in antigen‐presenting endothelial cells, Ta_4_C_3_T*_x_* MQDs have the mechanistic potential to reduce host inflammatory activation against allogeneic organs and tissues (Figure 6E). These Ta_4_C_3_T*_x_* MQDs are therefore promising materials for future applications in preventing allograft rejection and regenerative medicine.

### 2.8 Application of Ta_4_C_3_T*_x_* for In Vivo Immunomodulation

Finally, a rat model of allograft vasculopathy was used to explore the immunomodulatory effects of the synthesized Ta_4_C_3_T*_x_* MQDs in vivo. After solid organ transplantation, donor endothelial injury and activation results in the activation of alloreactive T‐lymphocytes in the recipient. One of the pathologic mechanisms for ultimate loss of the allograft is the development of allograft vasculopathy.^[^ ^66^, ^67^, ^68^ ^]^ This inflammatory condition uniquely manifests as accelerated narrowing of the blood vessels within transplanted hearts, lungs, and kidneys.^[^ ^69^, ^70^, ^71^ ^]^ Currently established treatments are largely ineffective and Ta_4_C_3_T*_x_* MXene‐based immunomodulation may offer promise as a novel therapy for this therapeutic challenge.

**Figure 7:**
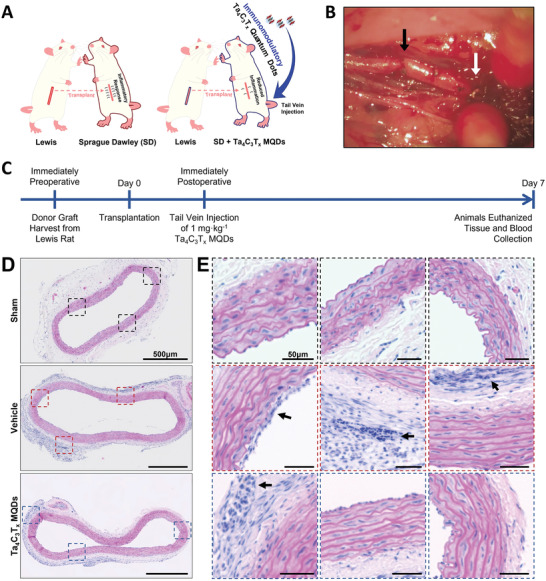
In vivo evaluation of the immunomodulatory effects of Ta_4_C_3_T*_x_* MQDs in a rat aortic allograft vasculopathy model. A) Schematic representation of the model. The descending thoracic aorta was transplanted from male Lewis rats into the abdomens of male Sprague‐Dawley rats. Animals received a tail‐vein injection of 1 mg kg^−1^ body weight of Ta_4_C_3_T*_x_* MQDs and were kept for 7 days. B) The photograph showing the transplanted aortic segment. Arrows represent the proximal (black) and distal (white) anastomoses. C) Experimental timeline for in vivo evaluation of Ta_4_C_3_T*_x_* MQDs. The donor graft was harvested immediately prior to the transplantation procedure and stored in ice‐cold saline until it was transplanted into the recipient. Tail‐vein injection of Ta_4_C_3_T*_x_* MQDs was performed immediately after the surgical procedure was completed. Blood and tissues were collected after 7 days of transplantation for further analysis. D,E) H&E staining of explanted abdominal aortic segments. Obvious signs of inflammation could be observed in the transplanted groups. Furthermore, there appeared to be quantitative reductions in the degree of endothelial thickening and adventitial lymphocyte infiltration in the MQD‐treated group when compared to the vehicle control (insets and arrows).

In the current study, the descending thoracic aorta was harvested from male Lewis rats and transplanted as an interposition graft into the abdominal aorta of male Sprague‐Dawley rats (**Figure** **7**A–C). Ta_4_C_3_T*_x_* MQDs at a dose of 1 mg kg^−1^ body weight (or an equivalent volume of saline, for control animals) were injected through the tail vein immediately after the transplantation. Animals were followed for one week after surgery, during which no adverse effects were observed with respect to the physical appearance, behavior, and body weight of animals. Blood and tissues were then collected for subsequent analysis. No gross histologic differences were noted in the lungs, liver, and kidneys between the treatment groups (Figure S12, Supporting Information). However, as shown in Figure 7D, histologic sections of the abdominal aorta from transplanted animals showed obvious inflammatory changes when compared with sham animals. Furthermore, significant differences were noted in both endothelial proliferation and adventitial immune cell infiltration between control and MQD‐treated animals (Figure 7E, arrows). Animals treated with intravenous Ta_4_C_3_T*_x_* MQDs appeared to have reduced endothelial injury and immune cell infiltration when compared with those injected with saline.

**Figure 8:**
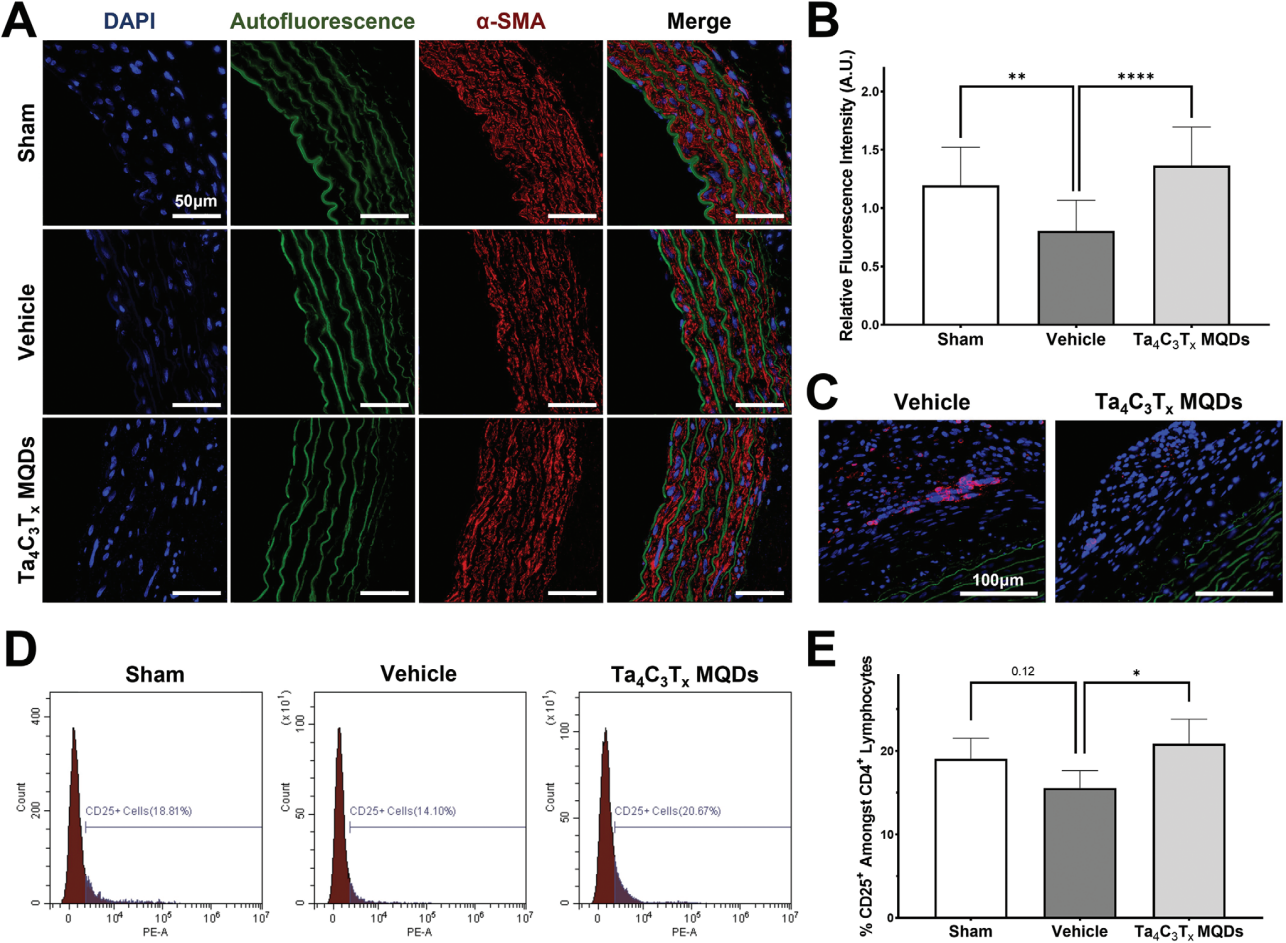
Quantitative assessment of the in vivo immunomodulatory effects of Ta_4_C_3_T*_x_* MQDs. A) Immunohistochemistry against α‐SMA showed significant disruption in the media of the transplanted aortic segments among transplanted animals, which was ameliorated with treatment using Ta_4_C_3_T*_x_* MQDs. B) α‐SMA was quantified using mean fluorescence intensity and normalized against a segment of the nontransplanted thoracic aorta from each animal. The vehicle control demonstrated a significant drop in the amount of α‐SMA, which was ameliorated with treatment using Ta_4_C_3_T*_x_* MQDs. C) Quantitative reductions were observed in the infiltration of CD8^+^ T‐lymphocytes (shown in red) in the adventitia of transplanted aortic segments between the Ta_4_C_3_T*_x_* MQD‐treated group when compared to the vehicle control group. D,E) Flow cytometric analysis of the circulating T‐lymphocytes of animals. Single lymphocytes were gated on the CD3^+^CD4^+^ gate. As shown here, there appeared to be lower numbers of CD25^+^ regulatory T‐lymphocytes in the aortic transplant group, which was ameliorated with treatment using 1 mg kg^−1^ body weight of intravenous Ta_4_C_3_T*_x_* MQDs.

To quantify the degree of vascular injury, immunohistochemistry was performed against alpha‐smooth muscle actin (α‐SMA), which is a marker for blood vessel integrity. An early sign of allograft vasculopathy is immunologic‐mediated loss of α‐SMA expressing medial smooth muscle cells.^[^ ^72^, ^73^ ^]^ Here, we noted a significant decrease in the amount of medial α‐SMA within the transplanted aortic segments of control animals (**Figure** **8**A). Furthermore, this loss of medial α‐SMA appeared to be ameliorated in animals treated with intravenous Ta_4_C_3_T*_x_* MQDs. When normalized against a segment of native thoracic aorta, transplanted aortic segments of treated animals displayed significantly better relative α‐SMA expression than control animals (Vehicle 0.8‐fold, MQDs 1.4‐fold, *p* < 0.0001; Figure 8B). Congruent with these observed changes, a higher number of infiltrating adventitial cytotoxic CD8^+^ T‐lymphocytes were observed within transplanted aortic segments of control animals when compared with those treated with Ta_4_C_3_T*_x_* MQDs (Figure 8C). Additionally, these findings were corroborated by flow cytometric identification of circulating CD4^+^CD25^+^ regulatory T‐lymphocytes (T_reg_s) performed one week after transplantation (Figure 8D,E). The T_regs_ are known to play a significant role in the development of immunologic tolerance after transplantation and higher numbers of T_regs_ is associated with reduced allograft vasculopathy after transplantation.^[^ ^74^ ^]^ In our study, transplanted animals had a numeric drop in the number of circulating T_regs_ (Sham 19.1%, Vehicle 15.5%, *p* = 0.12) when compared with sham animals, which was ameliorated by treatment with Ta_4_C_3_T*_x_* MQDs (Vehicle 15.5%, MQDs 20.9%, *p* < 0.05). This supports the proposed hypothesis that treatment with Ta_4_C_3_T*_x_* MQDs reduces immune activation, promotes allograft tolerance, and prevents immune‐mediated damage of transplanted allogeneic vascular segments. Taken together, these findings are highly suggestive of an in vivo immunomodulatory role for Ta_4_C_3_T*_x_* MQDs in the treatment of allograft vasculopathy.

## 3 Conclusion

In conclusion, the analysis within the current study presented the rational design, development, and application of immunoengineered tantalum carbide (Ta_4_C_3_T*_x_*) MXene quantum dots. As‐synthesized Ta_4_C_3_T*_x_* MQDs exhibited high concentrations of functional surface groups to facilitate their role in biomedical applications. Upon in vitro testing, these Ta_4_C_3_T*_x_* MQDs exhibited a direct interaction with human endothelial cells while maintaining excellent biocompatibility. In particular, Ta_4_C_3_T*_x_* MQDs are rapidly uptaken into ECs and reduce their ability to activate allogeneic T‐lymphocytes through regulation of surface coactivator and co‐inhibitor molecules. Additionally, when applied in an in vivo model of allograft vasculopathy, Ta_4_C_3_T*_x_* displayed strong immunomodulatory functions and reduced early development of allograft vasculopathy. This study for the first time highlights the strength and future potential of a rationally designed Ta_4_C_3_T*_x_* MQDs in immunoengineering and other biomedical applications.

## 4 Experimental Section

### Synthesis of Ta_4_C_3_T*_x_* MQDs

The 0D Ta_4_C_3_T*_x_* MQDs were synthesized from Ta_4_AlC_3_ MAX phase through etching, exfoliation, and subsequent hydrothermal process. First, bulky MAX phase was etched to synthesize 2D Ta_4_C_3_T*_x_* MXene nanosheets using HCl (216147, Fisher Scientific Co.) and NaF (≥99%, Sigma‐Aldrich). Briefly, Ta_4_AlC_3_ powder (Laizhou Kai Kai Ceramic Material Co., Ltd.) was slowly immersed and stirred in a mixture solution containing 12 m HCl and 4 m NaF at 60 °C for 48 h. The precipitated flakes were collected by high‐speed centrifugation, followed by several washing steps with pure distilled water at 10 000 rpm for 15 min each. The collected precipitates were freeze‐dried for 48 h and then dried in an air oven at 60 °C for 24 h. The resultant MXene nanosheets were further treated by bath sonication and probe homogenizer for 60 and 15 min, respectively, to obtain multi‐, oligo‐, and monolayer flakes, before being further treated by the hydrothermal process at 180 °C for 12 h. The collected aqueous MQDs suspensions were then sterilized using a steam autoclave and used for further experiments.

### Physicochemical Characterization of Ta_4_C_3_T*_x_* MQDs

Morphology and microstructural properties of materials were characterized using FESEM (SEM 450, Thermo Fisher Scientific), TEM (FEI Talos F200X S/TEM, Thermo Fisher Scientific), EDS, FTIR (Nicolet Nexus 870, Thermo Fisher Scientific), XPS (PHI Quantera, Physical Electronics, Inc.), and XRD (Bruker diffractometer). X‐ray diffraction peaks were collected in the range from 5° to 80° 2θ using a continuous scan with a rate of 3° min^−1^ and a report interval of 0.05°.

### Thermogravimetric and Optical Analysis

The TGA/DSC assessment of the Ta_4_C_3_T*_x_* MQDs was performed using a Q‐600 SDT (TA‐Instruments) on a DSC‐TGA Standard Module at a heating rate of 10 °C min^−1^ in air and argon (100 mL min). The temperature ramped up to 100 °C with a heating rate of 10 °C min^−1^, kept isothermal for 10 min, and ramped as high as 1000 °C. Furthermore, the optical properties of the aqueous Ta_4_C_3_T*_x_* MQDs suspensions at a concentration of ≈50 µg mL^−1^ were assessed by the Cytation5 Imaging Multi‐Mode Reader (BioTek) at different excitation–emission wavelengths.

### Zeta Potential Measurements

The surface charge of an aqueous Ta_4_C_3_T*_x_* MQDs colloidal suspension at a concentration of ≈75 µg mL^−1^ was assessed using the Nanobrook ZetaPALS (Brookhaven Instruments) at different pH of 2, 4, 6, 8, 10, and 12. The pH of the aqueous MQDs was titrated with the addition of adequate amounts of 12 m HCl and 12 m sodium hydroxide (NaOH) solutions. The electrical conductivity of aqueous Ta_4_C_3_T*_x_* MQDs was adjusted at same concentration using 0.1 phosphate‐buffered saline. The experiments were replicated for ten cycles, and the average values were reported.

### Animals and Ethics

All animal protocols were approved by the University of Manitoba Animal Care Committee and conform to standards and guidelines set out by the Canadian Council on Animal Care. Male Lewis rats (260–280 g) were used as donors and obtained from Charles River Laboratories. Male Sprague‐Dawley rats (260–280 g) were used as recipients and obtained from Central Animal Care Services at the University of Manitoba. All surgical procedures were performed at the R.O. Burrell Laboratory at the St. Boniface Hospital Research Centre, University of Manitoba, Winnipeg, according to standard operating procedures.

### Endothelial Cell Culture

Pooled human umbilical vein endothelial cells were obtained from Lonza (C2519A) and cultured in EGM‐2 (CC‐3162, Lonza) using manufacturer protocols unless otherwise specified. Briefly, cells were thawed in prewarmed EGM‐2 medium and seeded onto T‐25 flasks at a density of 2500 cells cm^−2^. The EGM‐2 media was changed 24 h after seeding and every 48 h thereafter. Cells were subcultured at 80% confluency using Trypsin (25200056, Gibco) and seeded into new vessels at 2500 cells cm^−2^. Prior to use, cells were further characterized to express the typical endothelial markers CD144 and vWF (Figure S13, Supporting Information). Cells used for experiments were between passages 3 and 5 for all experiments.

### Reactive Oxygen Species Assay

Total cellular ROS was assessed using the CellROX Green Reagent (C10444, Thermo Fisher Scientific). Briefly, HUVECs were plated on 96‐well plates and grown to 80% confluency. They were then treated subject to a nutrient starvation for 24 h by diluting the culture medium 1:1 with PBS in the presence of varying concentrations of MQDs. Cells were then imaged on a Nikon Ti‐2E fluorescence microscope and mean cellular fluorescence was quantified for 10 cells per high‐powered field using ImageJ software. Three replicates were included for each treatment condition.

### Caspase 3/7 Activity Assay

Caspase 3/7 activity was assessed using the CellEvent Caspase‐3/7 Green Detection Reagent (C10423, Thermo Fisher Scientific). Similar to the ROS assay, HUVECs were plated on 96‐well plates and grown to 80% confluency. The cells were then subjected to a nutrient starvation for 24 h by diluting the culture medium 1:1 with PBS in the presence of varying concentrations of MQDs. Subsequently, the cells were treated with Hoechst 33342 (R37605, Thermo Fisher Scientific) to define the nucleus and imaged on a Nikon Ti‐2E fluorescence microscope. Relative caspase activation was estimated based on the degree of nuclear fluorescence, with 10 nuclei quantified for each high‐powered field using ImageJ software. Three replicates were included for each treatment condition.

### Biocompatibility Assessment

Biocompatibility of the MQDs with HUVECs at 3 and 7 days was assessed using the Lactate Dehydrogenase (LDH) Cytotoxicity Detection Kit (MK401, Takara Bio). Briefly, HUVECs were plated on 96‐well plates and grown to 80% confluency. They were then treated with varying concentrations of MQDs and grown for 7 days in culture. At 3 and 7 days, media were taken from the wells for LDH assessment. Six replicates were included for each treatment condition. Additionally, cell proliferation at 7 days was assessed using the WST‐1 Cell Proliferation Assay kit (K304, BioVision Incorporated). Briefly, HUVECs were plated on 96‐well plates and grown to 80% confluency. The cells were then treated with varying concentrations of MQDs and grown for 7 days in culture and used for the WST‐1 assay according to manufacturer protocols. Five replicates were included for each treatment condition.

### Assessment of Cellular Uptake

HUVECs were plated on chamber slides and cultured with MQDs at a concentration of 20 µg mL^−1^ for 24 h. The cells were then fixed with 4% paraformaldehyde and mounted using ProLong Diamond Antifade Mountant with DAPI (P36962, Thermo Fisher Scientific). The fixed cells were imaged on a Nikon Ti‐2E fluorescence microscope using the bright‐field mode and the DAPI filter. The images showing cellular uptake are presented in the manuscript.

### Western Blot Analysis

Western blot analysis was used to confirm the induction of major histocompatibility complex‐II (MHC II) expression. Briefly, HUVECs were treated with or without 20 µg mL^−1^ of Ta_4_C_3_T*_x_* MQDs for 24 h prior to induction with 10 units mL^−1^ of IFN‐γ. Cells were then scraped on ice and collected in Radio Immunoprecipitation Assay (RIPA) Buffer. The cell lysate was spun at 12 000 × *g* at 4 °C for 10 min to collect the supernatant. The total protein in the supernatant was then quantified using the Bradford Protein Quantification Assay (5000006, Bio‐Rad). For gel electrophoresis, 30 µg of protein was loaded into a 10% polyacrylamide resolving gel (1610658, Bio‐Rad) and run at 100 V for ≈2 h. The protein was then transferred onto Polyvinylidene Fluoride (PVDF) membrane at 4 °C for 12 h. Membranes were blocked with 5% skim milk for 1 h at room temperature and probed using the desired primary antibodies overnight at 4 °C. Membranes were then washed and secondary antibody was added for 1 h at room temperature. The signal was detected using Pierce ECL Western Blotting Substrate (32209, Thermo Fisher Scientific) and a ChemiDoc MP (Bio‐Rad). Signal intensities were normalized to beta‐actin and quantified using Quantity One 1D Analysis Software (Bio‐Rad, Hercules, California). The list of primary and secondary antibodies used for this experiment and their dilutions are presented in Table S2 in the Supporting Information.

### Immunomodulation Assays

HUVECs were plated on 24‐well plates at a density of 20 000 cells per well and allowed to attach for 24 h. The cells were then treated with MQDs at 2 µg mL^−1^ for 24 h. Subsequently, HUVECs were activated using IFN‐γ (570202, BioLegend) at a concentration of 10 units mL^−1^ for 24 h. Cells were then washed in preparation for subsequent coculture experiments. Human PBMNCs were isolated from whole blood obtained from healthy volunteers using Lympholyte‐H Cell Separation Media (CL5015, Cedarlane Labs). Cocultures were performed in EGM‐2 supplemented with 5 ng mL^−1^ of interleukin‐2 (589102, Biolegend) and varying concentrations of MQDs for 9 days. At this point, cells were pulsed with the Cell Stimulation Cocktail with protein transport inhibitor (00‐4975‐93, Thermo Fisher Scientific) for 6 h, after which PBMNCs were collected for subsequent flow cytometric analysis.

In a separate experiment, the direct immunomodulatory effects of Ta_4_C_3_T*_x_* MQDs were investigated in the absence of HUVECs. Briefly, naïve CD4^+^ T‐lymphocytes were isolated through negative magnetic activated cell sorting using the MojoSort Human CD4 Naïve T Cell Isolation Kit (480041, BioLegend). Cells were cultured in 24‐well plates at a density of 10^5^ cells per well and stimulated with 10 µg mL^−1^ of plate‐bound anti‐CD3 antibody (300313, BioLegend) and 2 µg mL^−1^ of soluble anti‐CD28 antibody (302913, BioLegend) at the start of culture immediately after isolation. Cells were grown in Advanced RPMI 1640 medium (12633012, Gibco) supplemented with 10% FBS (12483020, Gibco), 2 × 10^−3^ m GlutaMAX (35050061, Gibco), 1:100 penicillin–streptomycin (15140122, Gibco), 0.055 × 10^−3^ m 2‐mercaptoethanol (M3148, Sigma‐Aldrich), and 20 units mL^−1^ recombinant human IL‐2 (589102, BioLegend). For T_H_1 polarization, the medium was also supplemented with 10 ng mL^−1^ recombinant human IL‐12 (573002, BioLegend). The cells were cultured for one week and analyzed using flow cytometry.

### Flow Cytometry

The list of flow cytometry antibodies and the concentrations used are presented in Table S3 in the Supporting Information. Briefly, cells were collected after the aforementioned experiments in ice‐cold Flow Cytometry (FACS) buffer consisting of phosphate‐buffered saline, 1% bovine serum albumin, 2 × 10^−3^ m EDTA, and 0.1% sodium azide. Cells were then fixed and permeabilized using the eBioscience Staining Buffer Set (00‐5523‐00, Thermo Fisher Scientific) and stained for 1 h at room temperature using manufacturer recommended antibody concentrations. Prior to analysis, cells were washed once in FACS buffer and resuspended in 100 µL of FACS buffer. Cells were analyzed on the CytoFLEX Flow Cytometer (Beckman Coulter) with the appropriate fluorescence‐minus‐one and isotype controls. Data analysis was performed using CytExpert Software version 2.3.1.22 (Beckman Coulter, Brea, California).

For analysis of in vivo samples, live cell staining was performed. Briefly, peripheral blood mononuclear cells were collected from blood using a Ficoll density gradient (Histopaque‐1083, 10831, Sigma‐Aldrich). Cells were then washed in ice‐cold FACS buffer and stained for 1 h at 4 °C using manufacturer recommended antibody concentrations. Prior to analysis, cells were washed in ice‐cold FACS buffer and resuspended in 100 µL of FACS buffer. The eBioscience 7‐AAD Viability Staining Solution was used to exclude nonviable cells (00‐6993‐50, Thermo Fisher Scientific). Cells were analyzed on the CytoFLEX Flow Cytometer with the appropriate fluorescence‐minus‐one and isotype controls. Data analysis was performed using CytExpert Software version 2.3.1.22.

### Quantitative PCR Analysis

The list of primers used for quantitative PCR is presented in Table S4 in the Supporting Information. Total cellular RNA was isolated using the Aurum Total RNA Mini Kit (7326820, Bio‐Rad) and quantified using a NanoDrop Spectrophotometer (Thermo Fisher Scientific). cDNA was synthesized using the High‐Capacity cDNA Reverse Transcriptase Kit (4368814, Thermo Fisher Scientific) using the manufacturer recommended protocol. qPCR was performed using the CFX384 Touch Real‐Time PCR Detection System (Bio‐Rad) with the appropriate no template controls.

### In Vivo Aortic Transplantation Model

Animal care and anesthesia was performed using standard operating procedures at the University of Manitoba. After induction of anesthesia, donor Lewis rats underwent a median sternotomy where the mediastinal structures were removed. Immediately afterward, the thoracic aorta was mobilized and harvested, taking care to mark the proximal end and to ligate branch vessels to ensure subsequent hemostasis. The donor aorta was stored in ice‐cold saline for subsequent transplantation. Recipient animals underwent induction of anesthesia and median laparotomy. The abdominal viscera were displaced to access the retroperitoneum. The infrarenal abdominal aorta was isolated using careful dissection and branch vessels inferior to the gonadal arteries were ligated. Next, clamps were placed on the abdominal aorta and a segment was resected. The previously harvested donor thoracic aortic segment was then anastomosed as an interposition graft using two end‐to‐end anastomosis with 8‐0 Prolene sutures. Hemostasis was ensured using a combination of pressure and SurgiCel. Good pulses were appreciated in the distal segment of the aorta prior to closure. The abdominal viscera was then replaced into the abdomen and the abdomen was closed in a routine fashion. Animals were kept for one week, after which point the transplanted aortic segment as well as a segment of the thoracic aorta were harvested from each animal for subsequent analysis. Blood was also collected at the time of harvest for flow cytometry.

### Immunohistochemistry

The list of antibodies used for immunohistochemistry is presented in Table S5 in the Supporting Information. After collection, tissues were fixed in 10% buffered formalin (SF100, Fisher Scientific) at room temperature overnight. The tissues were then embedded in paraffin blocks and sectioned to 5 µm thick sections and mounted on glass slides. Hematoxylin and eosin staining was performed in a regressive fashion with Harris’ hematoxylin using standard protocols. For immunohistochemistry, sections were rehydrated using a series of ethanol steps and then incubated overnight with primary antibody at 4 °C. The tissue sections on slides were then washed and incubated with secondary antibody for 1 h at room temperature. Finally, the sections were mounted with Prolong Diamond Antifade Mountant with DAPI and imaged using a Nikon Ti‐2E fluorescence microscope.

### Statistical Analysis

Comparisons between two variables were performed using an unpaired two‐tailed Student's *t*‐test. Comparison between three or more groups were performed using a one‐way analysis of variance (ANOVA) and Tukey's honestly significant difference test. Comparisons involving two independent variables were compared using a two‐way analysis of variance and the Bonferroni multiple comparisons test. An adjusted *p*‐value less than 0.05 was considered to be significant. All statistical analysis was performed using Prism version 9.02 for Windows (GraphPad, San Diego, California).

## Conflict of Interest

R.C.A. has received an unrestricted educational grant from Pfizer Canada Inc. and honoraria from AVIR Pharma Inc., Abbott Nutrition, and Edwards Lifesciences for work unrelated to this work. All authors declare no conflict of interest.

## Author Contributions

A.R. and W.Y. contributed equally to this work. The study was conceptualized and designed by A.R., W.Y., and S.D. A.R., W.Y., K.N.A., A.S., and N.S. carried out the experiments and acquired the data. A.R., W.Y., R.C.A., and S.D. interpreted the data and performed statistical and formal analysis. A.R., W.Y., and S.D. designed the figures and drafted the manuscript. All authors read and approved the final manuscript.

## Supporting information

Supporting Information

## Data Availability

The data that support the findings of this study are available from the corresponding author upon reasonable request.
