# Supplementary material for: Fabrication of Smart Tantalum Carbide MXene Quantum Dots with Intrinsic Immunomodulatory Properties for Treatment of Allograft Vasculopathy
Source: Adv Funct Mater. 2021 Sep 8;31(46):2106786. doi: 10.1002/adfm.202106786 (PMC8820728; doi:10.1002/adfm.202106786)
Supplement: Supplementary file 1 — Supporting Information [file ADFM-31-0-s001.pdf]

© 2021 Wiley-VCH GmbH

ADVANCED FUNCTIONAL MATERIALS

Supporting Information

for *Adv. Funct. Mater.,* DOI: 10.1002/adfm.202106786

Fabrication of Smart Tantalum Carbide MXene Quantum Dots with Intrinsic Immunomodulatory Properties for Treatment of Allograft Vasculopathy

*Alireza Rafieerad, Weiang Yan, Keshav Narayan Alagarsamy, Abhay Srivastava, Niketa Sareen, Rakesh C. Arora, and Sanjiv Dhingra**

Electronic Supplementary Information

Fabrication of Smart Tantalum Carbide MXene Quantum Dots with Intrinsic Immunomodulatory Properties for Treatment of Allograft Vasculopathy

Alireza Rafieerad[a,b,#], Weiang Yan[a,b,c,#], Keshav Narayan Alagarsamy[a,b], Abhay Srivastava[a,b], Niketa Sareen[a,b], Rakesh C. Arora[b,c], Sanjiv Dhingra[a,b,*]

[a] *Regenerative Medicine Program, Department of Physiology and Pathophysiology, Rady Faculty of Health Sciences, University of Manitoba, Winnipeg, Manitoba, R3E 0W2, Canada*

[b] *Institute of Cardiovascular Sciences, Albrechtsen St. Boniface Research Centre, University of Manitoba, Winnipeg, Manitoba, R2H 2A6, Canada*

[c] *Section of Cardiac Surgery, Department of Surgery, Max Rady College of Medicine, Rady Faculty of Health Sciences, University of Manitoba, Winnipeg, Manitoba, R3E 0W2, Canada*

[#] *A.R and W.Y contributed equally to this work.*

***Correspondence:**
Sanjiv Dhingra, PhD, FAHA, FAPS
Regenerative Medicine Program
Director: Canada Italy Tissue Engineering Program
Institute of Cardiovascular Sciences, St. Boniface Hospital Research Centre
R-3028-2, 351 Tache Avenue, Winnipeg, R2H2A6, Canada
Email: sdhingra@sbrc.ca

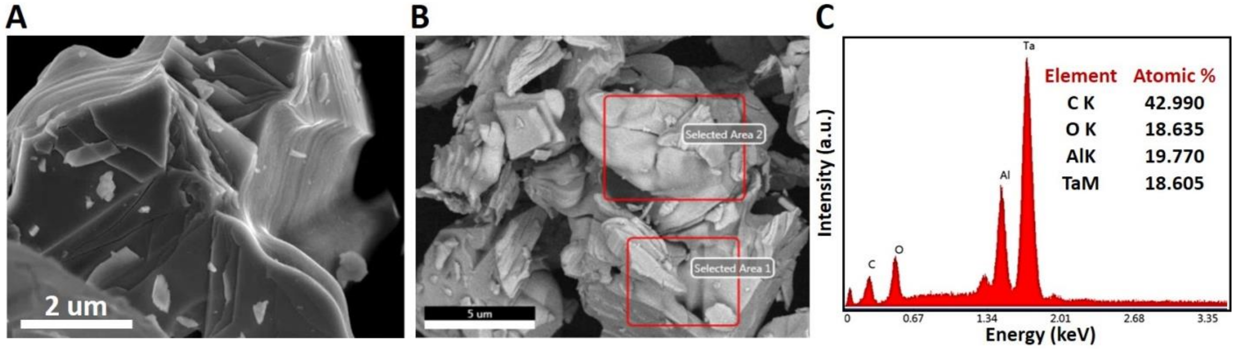

Supplementary Figure S1*: Morphology and elemental composition characterization of Ta_4AlC_3 MAX phase.* (**A**) FESEM image showing the microstructure of Ta_4AlC_3 MAX phase bulk before etching with the HCl/NaF. (**B**, **C**) Back-scattered SEM image and EDS analyses of Ta_4AlC_3 MAX phase to evaluate its elemental composition. The EDS histogram revealed C, Ta, O, and Al as the main elements. In particular, EDS results confirmed significant Al layers in the material structure, with an atomic percentage of ~ 20%.

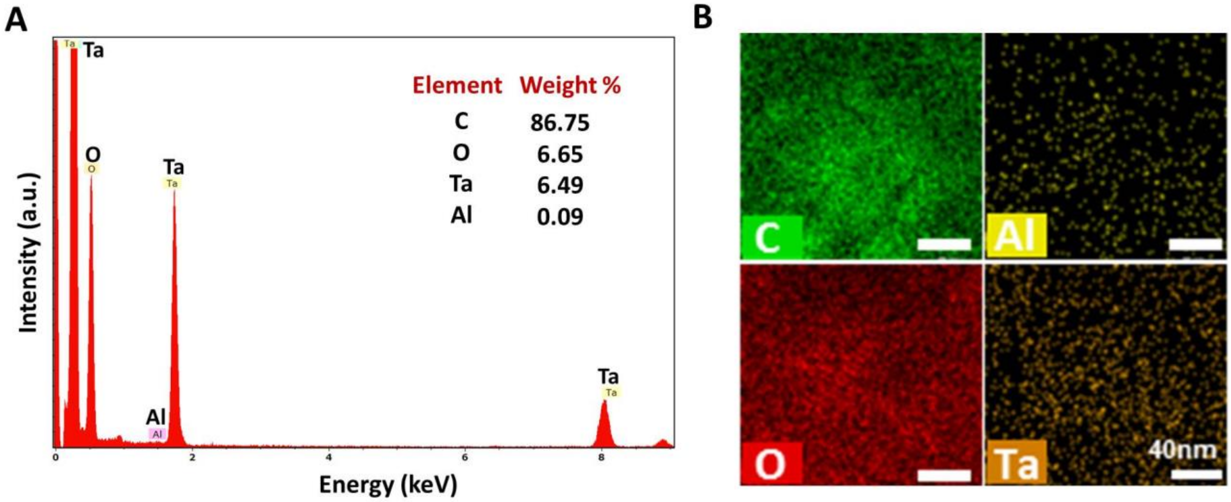

Supplementary Figure S2: ***Elemental composition analysis of the HCl/NaF exfoliated $Ta_4C_3T_x$ MXene nanosheets.*** (**A**) EDS histogram showed C, Ta, and O as the main elemental composition of the etched nanosheets. The weight percentage of Al (~ 0.1%) confirmed that our innovative protocol could etch and exfoliate the Ta_4AlC_3 MAX phase to MXene nanosheets. (**B**) EDS mapping further demonstrated the distribution of C, Ta, and O within the $Ta_4C_3T_x$ MXene nanosheets.

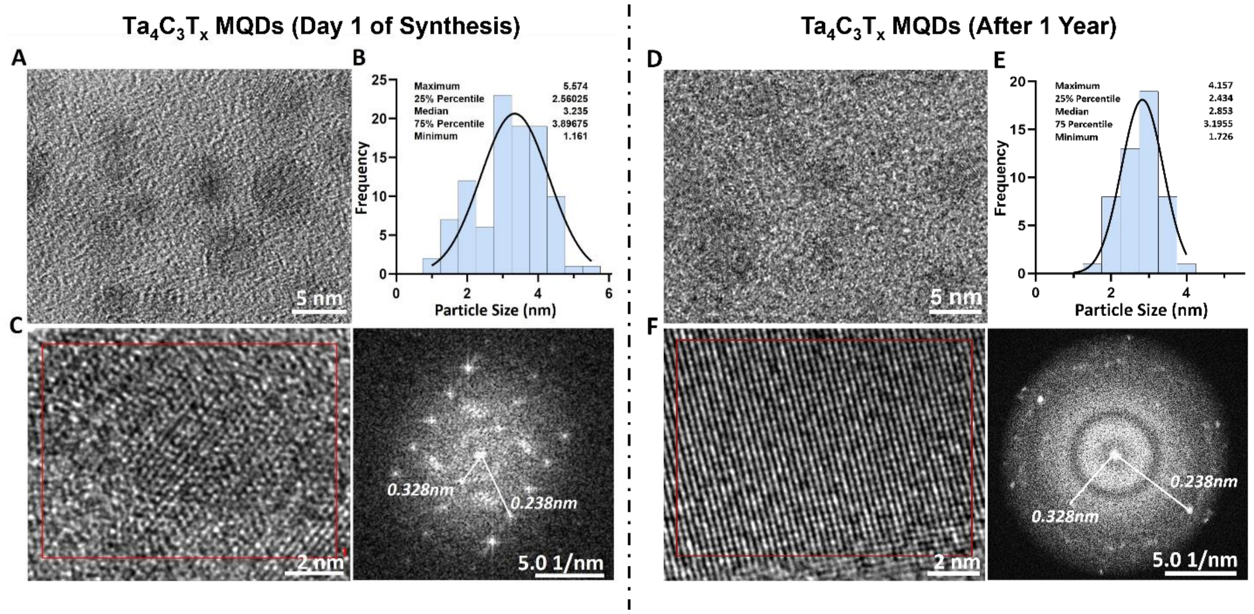

Supplementary Figure S3: ***Size and crystallinity of $Ta_4C_3T_x$ MQDs.*** (**A**, **B**) High-resolution TEM (HRTEM) micrograph of MQDs revealed uniform distribution of particles with a median size of 3.323 nm. The statistical analysis and frequency histogram revealed that the maximum particle size of a single MQD is 5.574 nm. This geometry was specifically obtained by the applied hydrothermal treatment and can be varied at different processing temperatures. (**C**) The HRTEM image and corresponding fast Fourier transform (FFT) pattern for a single $Ta_4C_3T_x$ MQD demonstrated a highly crystalline 0D material with lattice d-spacing of 0.328 nm (inner plane: 0.238 nm). Our FFT data illustrate the orientation of the MQDs that demonstrates the crystalline structure of the material. (**D-F**) HRTEM and FFT of $Ta_4C_3T_x$ MQDs at 1 year after synthesis revealed no significant changes compared to the initial measurements, confirming the long-term stability and shelf-life of as-synthesized quantum dots.

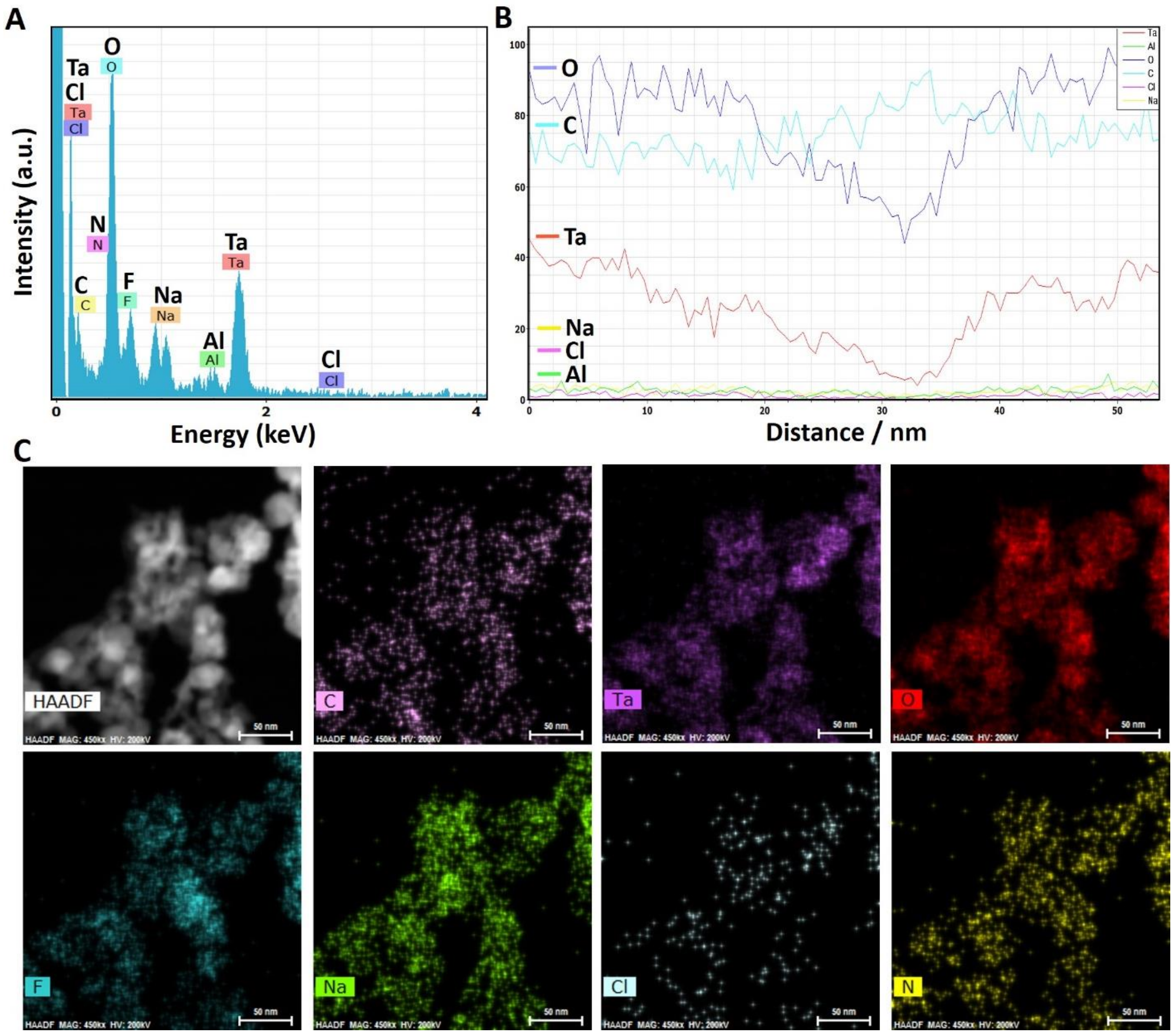

Supplementary Figure S4: ***Elemental composition analysis of as-synthesized $Ta_4C_3T_x$ MQDs.*** (**A**) The EDS point and (**B**) line scan analysis of MQDs showed that the main elemental composition of the particles included Ta, C, O, F, and Na. This suggested that the employed fabrication method in the current study was effective, and the Al layers were significantly removed during the synthesis process. Also, the low concentration of Cl in the MQDs composition confirms the efficiency of HCl to synthesize MXene materials. The relatively higher concertation of Na and F in elemental map describes the bonding in MQDs structure. (**C**) The scanning TEM image and EDS mapping of MQDs further showing the elemental composition.

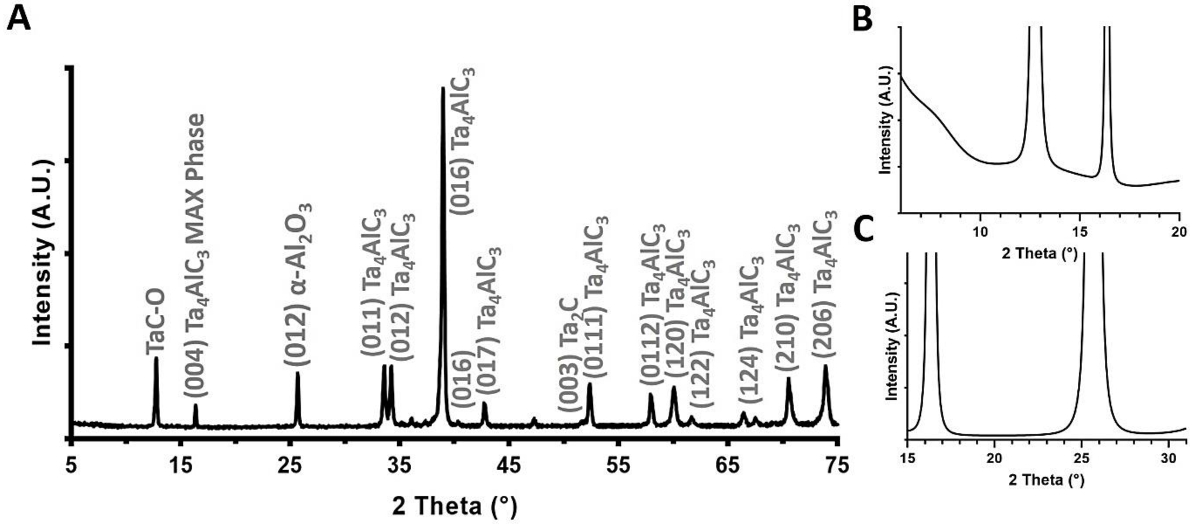

Supplementary Figure S5: ***XRD phase characterization of pristine*** $\boldsymbol{Ta_4AlC_3}$ ***MAX phase.*** (**A-C**) The XRD analysis of bulky Ta_4AlC_3 MAX phase revealed its crystalline structure with significant presence of Al. The XRD spectra of MAX phase contains a dominant peak at ~ 16º 2θ. The phase pattern of Ta_4AlC_3 includes a contamination peak of tantalum carbide (Ta_2C) at ~ 50º 2θ. These data are in agreement with the standard XRD structure of Ta_4AlC_3 MAX phase. The peaks of the tantalum aluminium carbide were matched with a standard XRD pattern (ICSD156383, 96-210-3218, α-alumina).

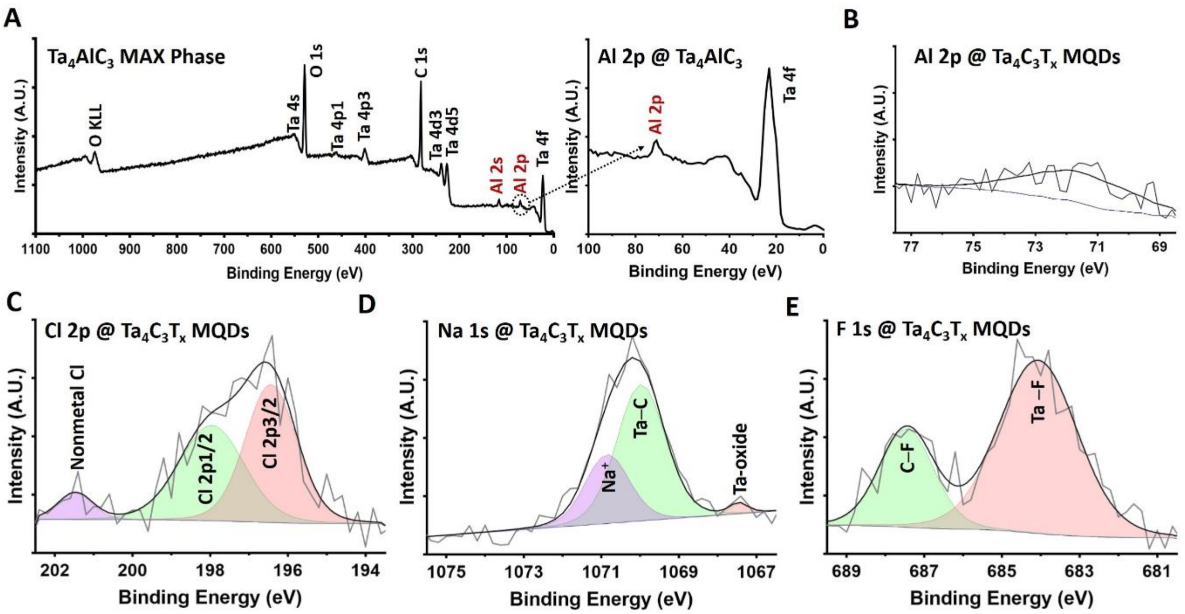

Supplementary Figure S6: ***XPS characterization of Ta_4AlC_3 MAX phase and $Ta_4C_3T_x$ MQDs.*** (**A**) Wide scan survey of the MAX phase showing its surface composition. The XPS measurement detected a significant amount of Al in MAX phase with an atomic percentage of 3.19. The presence of atomic oxygen in the MQDs increased from 33.15% in MAX phase to 43.51% in MQDs (**Figure 2E**). (**B**) The Al 2p XPS narrow scan spectrum of $Ta_4C_3T_x$ MQDs displayed significant removal of Al from the material. (**C-E**) The Cl 2p, Na 1s, and F 1s spectra of $Ta_4C_3T_x$ MQDs. The F 1s include Ta–F, and C–F bonds at the binding energies of 684.06 and 687.45. This provided further support that our innovative protocol successfully synthesized, and surface functionalized the MQDs.

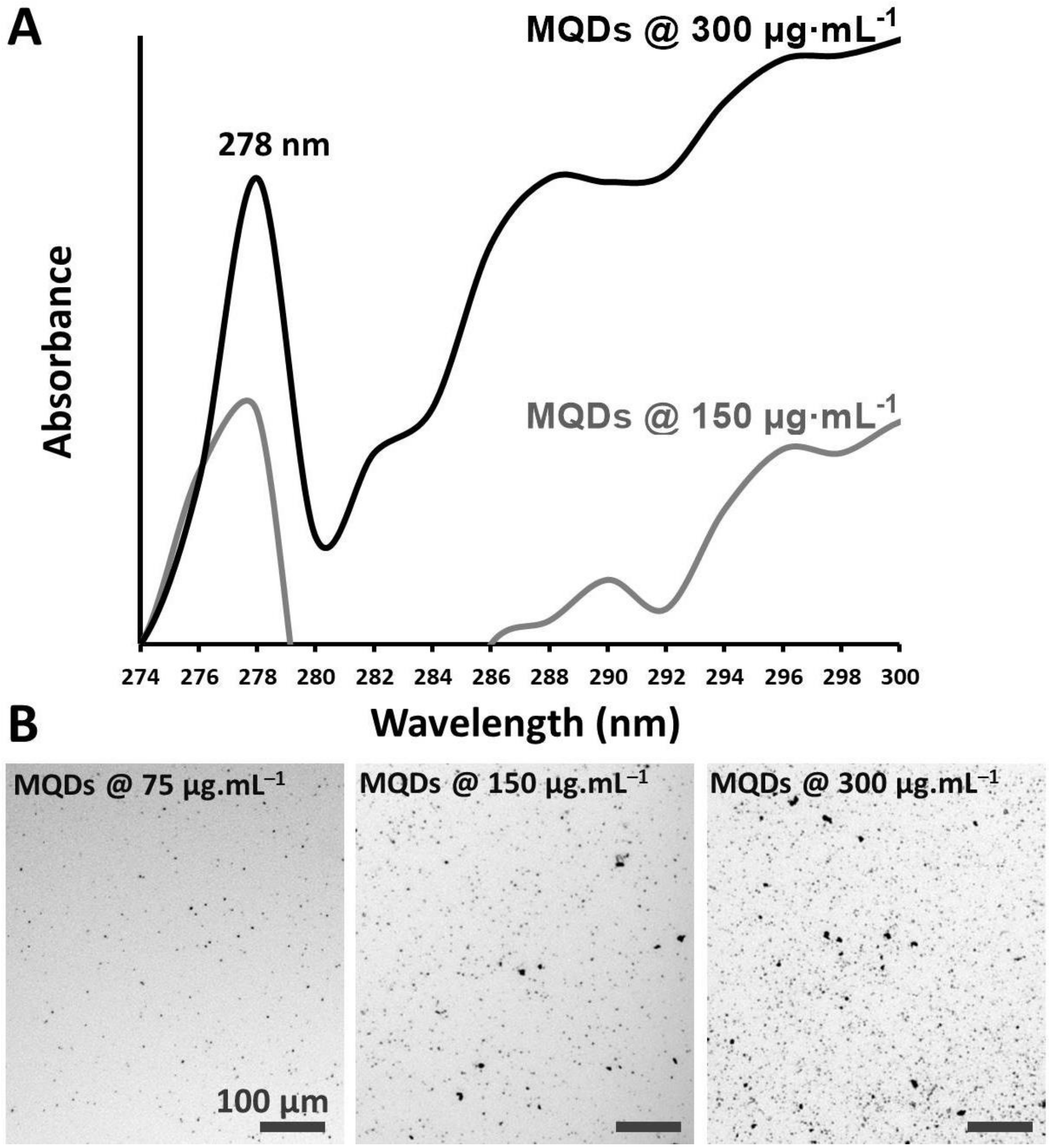

Supplementary Figure S7: ***Assessment of optical absorption of $Ta_4C_3T_x$ MQDs.*** (**A**) Our UV-Vis spectrum of MQDs displayed dose-dependent absorption properties with a broad peak at a wavelength of ~ 300 nm. Additionally, when the UV-Vis analysis of $Ta_4C_3T_x$ MQDs was repeated six months after synthesis, no significant differences were observed in its optical absorption profile. This confirmed adequate stability of as-synthesized MQDs for the targeted biomedical applications. (**B**) Furthermore, optical microscope images of colloidal $Ta_4C_3T_x$ MQDs showed high stability in aqueous media without significant agglomeration or precipitation at concentrations up to 300 µg·mL^{-1}.

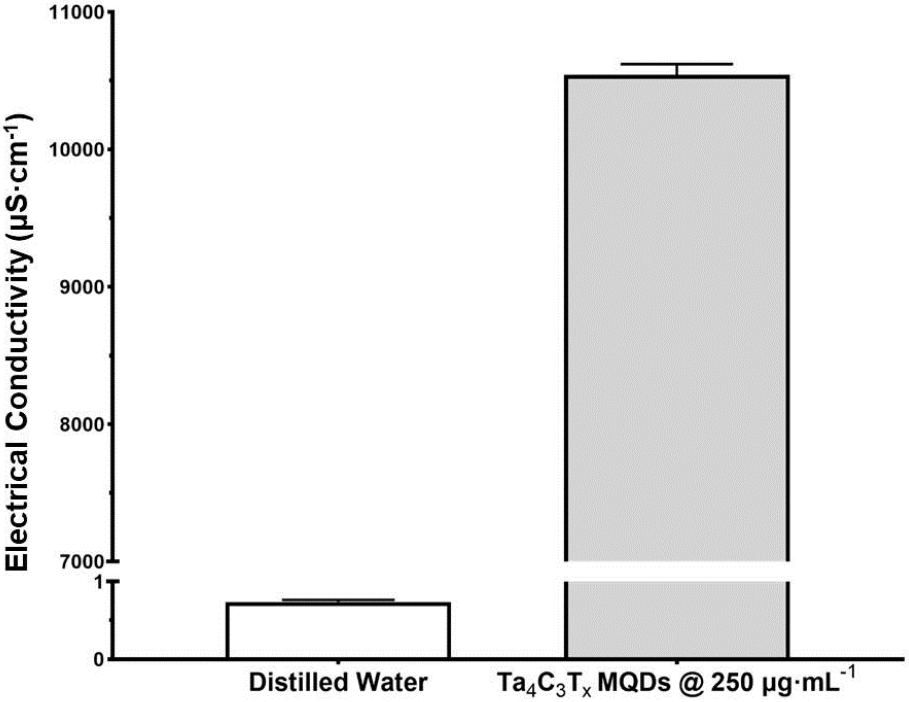

Supplementary Figure S8: ***Assessment of electrical conductivity of $Ta_4C_3T_x$ MQDs.*** The electrical conductivity of $Ta_4C_3T_x$ MQDs as measured by DuraProb 4-Electrode Probe (Thermo Scientific) in an aqueous colloidal dispersion was 10543 ± 77 μScm^{-1} at a concentration of 250 μgmL^{-1}, compared to 0.737 ± 0.027 μScm^{-1} for distilled water measured under the same conditions.

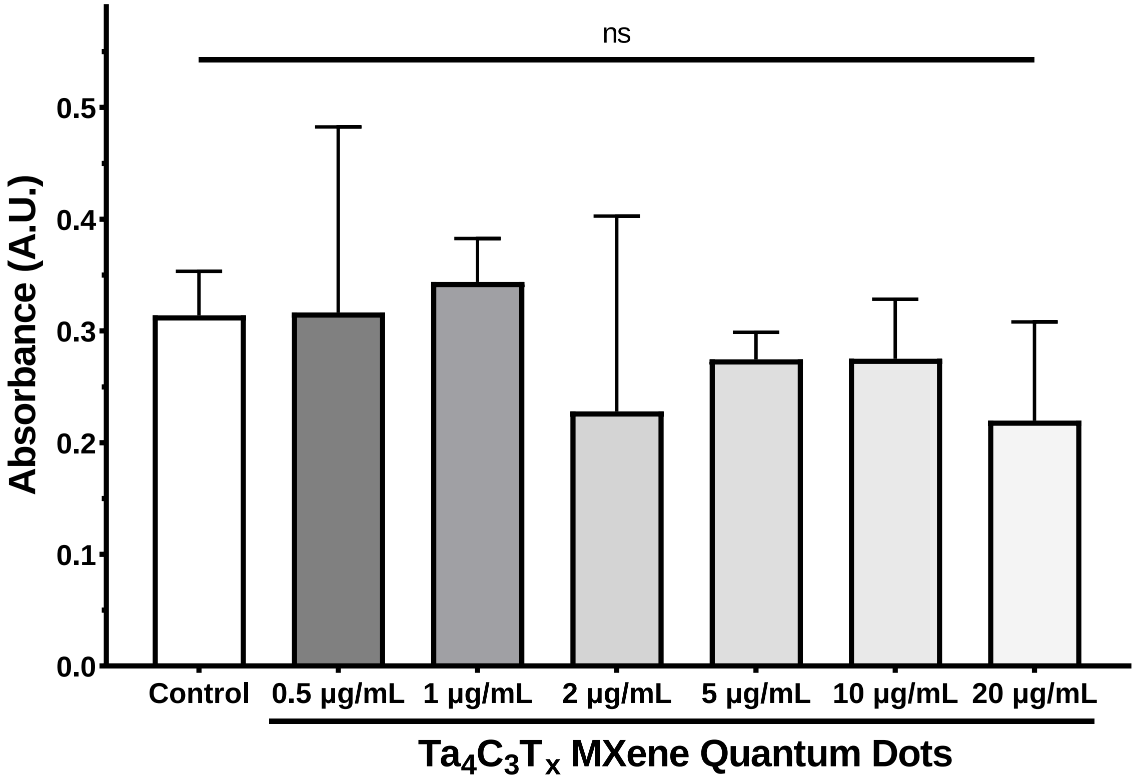

Supplementary Figure S9: ***Assessment of lactate dehydrogenase release from endothelial cells cultured with $Ta_4C_3T_x$ MQDs at 7 days.*** No significant differences were observed in cytotoxicity between control cells and those treated with 0.5 to 20 µg·mL^{-1} of $Ta_4C_3T_x$ MQDs.

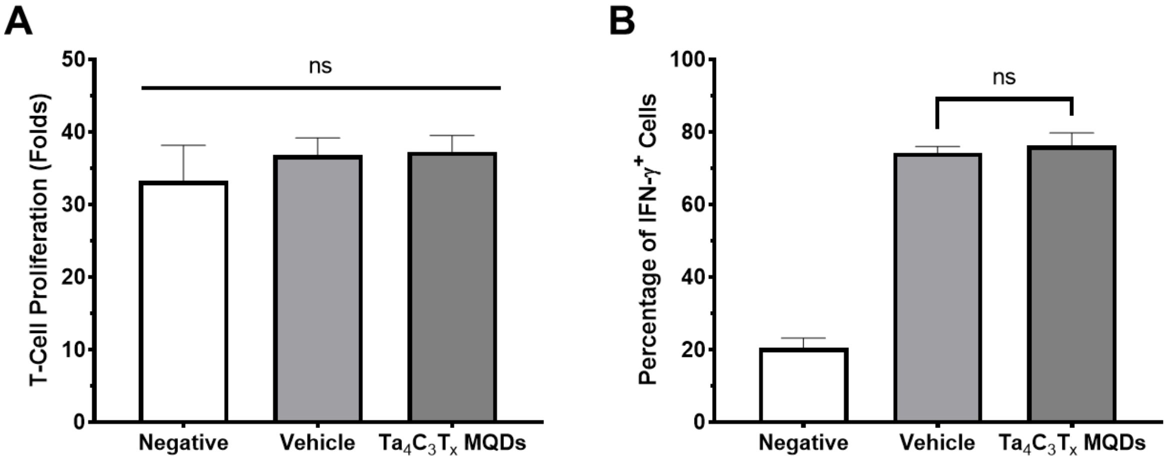

Supplementary Figure S10: ***Co-culture of activated and polarized naïve human CD4$^+$ lymphocytes with $Ta_4C_3T_x$ MQDs.*** Naïve human CD4$^+$ T-lymphocytes were isolated using negative magnetic activated cell sorting and activated/polarized using plate-bound anti-CD3, soluble anti-CD28, and exogeneous IL-2 and IL-12 in the presence and absence of $Ta_4C_3T_x$ MQDs. (**A**) After 7 days, no significant differences were observed in the degree of T-cell proliferation and (**B**) percentage of T_H1 (IFN-γ^+) CD4$^+$ T-lymphocytes between cells treated with and without $Ta_4C_3T_x$ MQDs.

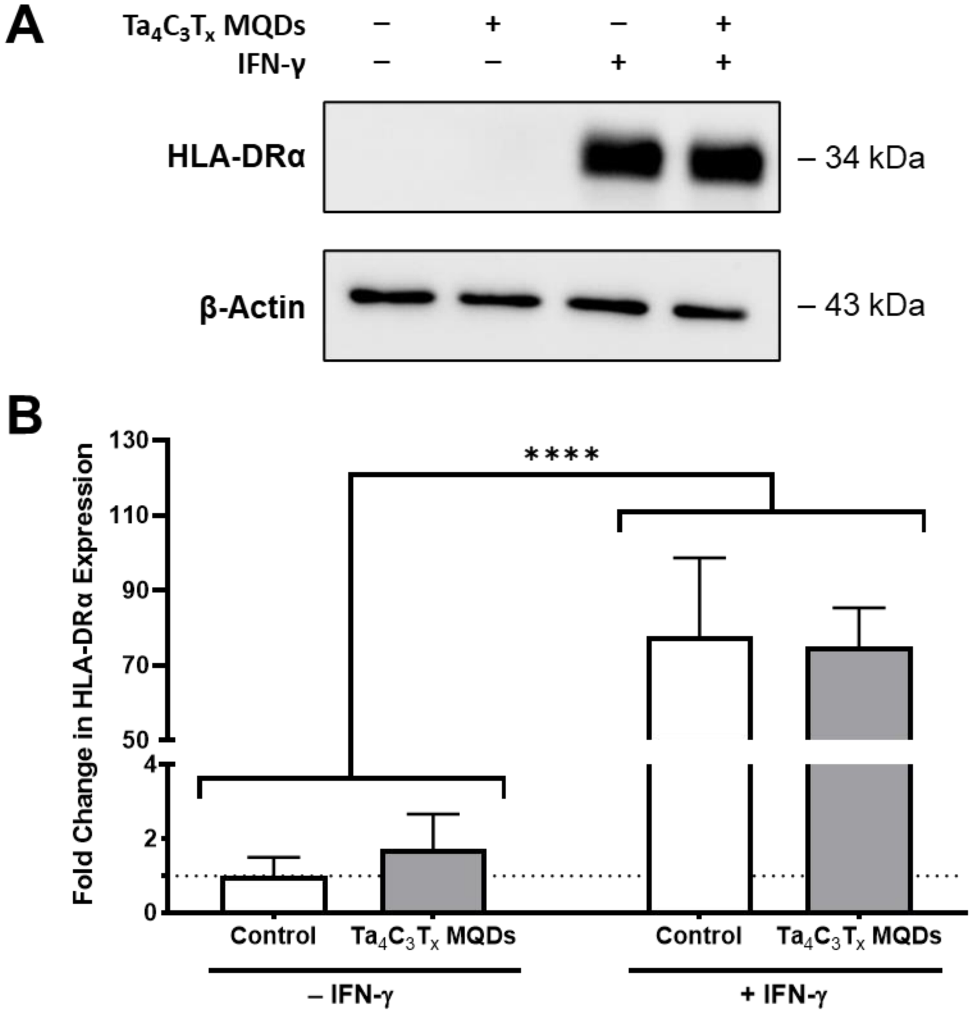

Supplementary Figure S11: ***Activation of human umbilical vein endothelial cells by interferon gamma (IFN-γ) after 24 hours.*** HLA-DRα expression was normalized to β-actin expression in order to assess endothelial activation and antigen presentation induced by IFN- γ. (**A-B**) As shown by Western Blot analysis, addition of 10 units·mL^{-1} of recombinant IFN-γ induced robust activation of human umbilical vein endothelial cells in both control cells and those treated with 20 µg·mL^{-1} of $Ta_4C_3T_x$ MQDs ($p<0.001$ for the comparison shown).

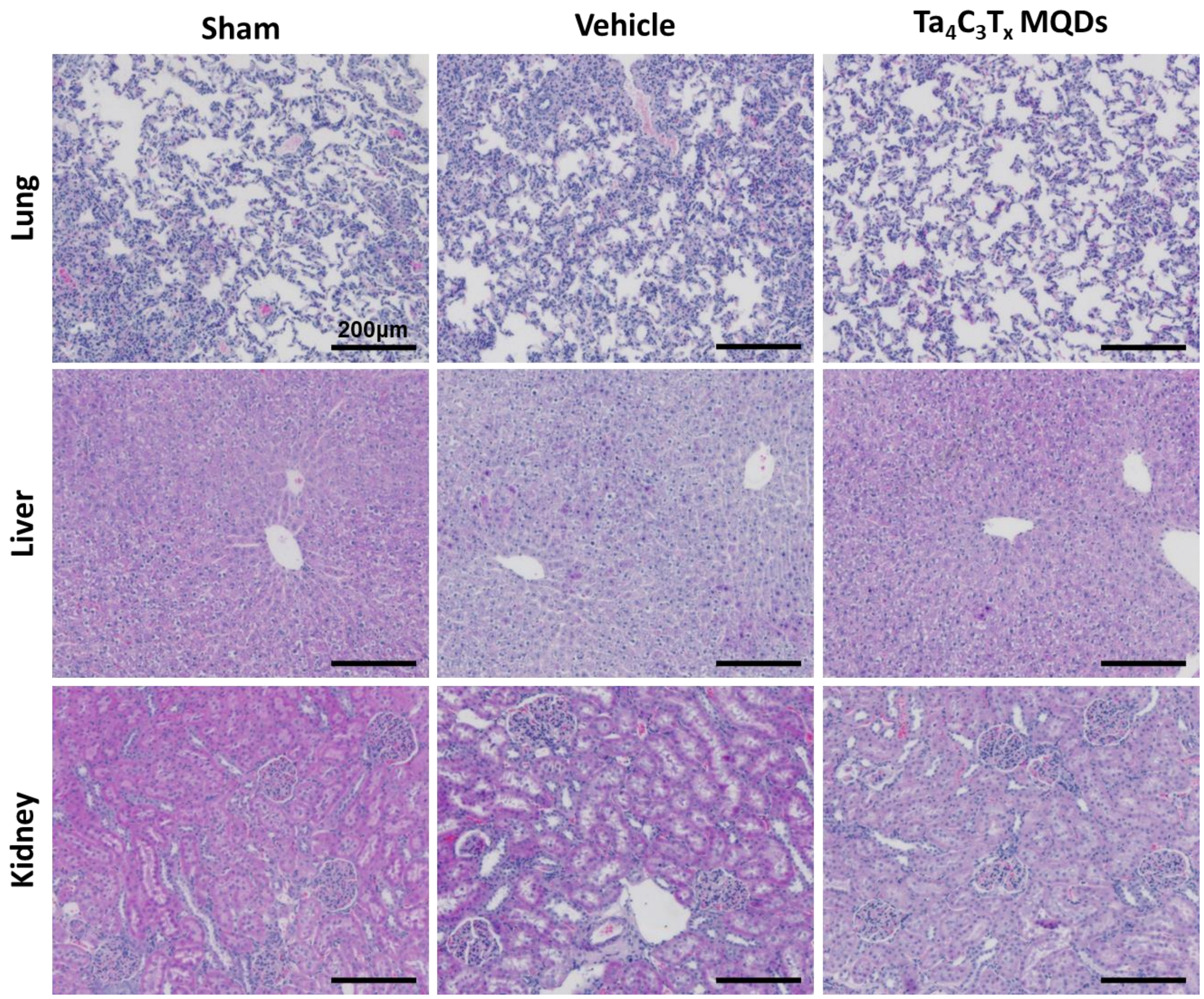

Supplementary Figure S12: ***Histologic assessment of the lung, liver, and kidney from animals undergoing aortic transplantation and $Ta_4C_3T_x$ MQDs injection.*** No gross histologic changes were observed on H&E staining of sections from these organs.

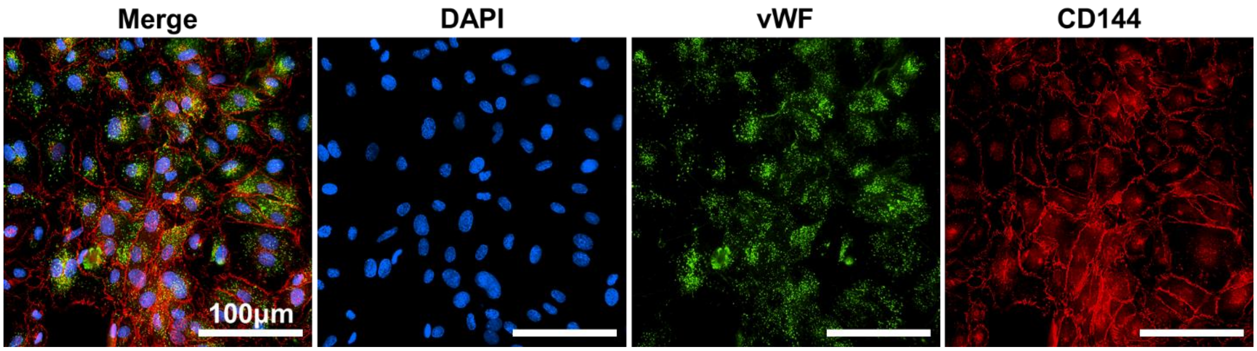

Supplementary Figure S13: ***Characterization of human umbilical vein endothelial cells used for in vitro experiments.*** Cells were characterized by the commercial vendor as 100% double positive for PECAM-1 (CD31) and Endoglin (CD105) and were additionally tested in our study to be strongly positive for both von Willebrand factor (vWF) and VE-Cadherin (CD144). This is in keeping with the expected surface marker expression for HUVECs.

Supplementary Table S1: ***XPS peak fitting results for $Ta_4C_3T_x$ MQDs.*** The XPS wide scan atomic percentage of $Ta_4C_3T_x$ MQDs and its Ta_4AlC_3 MAX phase. Peak fitting results for $Ta_4C_3T_x$ MQDs are also presented here.

Sample	Elements	Overall Atomic %	Component Name	Binding Energy (eV)	Component Atomic %	FWHM (eV)
$Ta_4C_3T_x$ MQDs	Ta $4f_{7/2}$ ($4f_{5/2}$)	9.72	Ta–C_x	21.57	1.2	2.37
			Ta^{4+}–O ($TaO_{2\ 7/2}$)	24.49	32.06	1.29
			Ta–C_x	24.89	30.6	2.25
			Ta^{4+}–O ($TaO_{2\ 5/2}$)	26.31	17.4	1.15
			Ta^{5+}–O ($Ta_2O_{5\ 7/2}$)	26.78	18.13	1.48
			Ta^{5+}–O ($Ta_2O_{5\ 5/2}$)	28.79	0.61	0.78
	C 1s	40.77	C–Ta	281.22	0.09	1.02
			C–Ta	283.23	45.81	1.31
			C=C	283.76	13.89	1.77
			C–C	285.12	32.39	1.33
			C–N	286.22	4.58	0.98
			C–O	287.02	3.16	1.69
			C=O	288.7	0.08	0.62
	O 1s	43.51	Ta–Oxide	528.55	9.07	1.41
			$Ta_4C_3O_x$	529.66	8.29	1.61
			$Ta_4C_3(OH)_x$	531.42	81.1	1.99
			H_2O_{ads} / $Ta_4C_3(OH)_x$	534.6	1.53	1.79
	F 1s	2.42	Ta–F	684.06	71.82	2.39
			C–F	687.45	28.18	1.65
	Cl $2p_{3/2}$ ($2p_{1/2}$)	1.81	Cl 2p	196.44	88.46	1.56
			Nonmetal Cl	201.48	11.54	1.06
	Na 1s	1.35	Ta–Oxide	1067.45	1.86	0.54
			Ta–C	1069.97	67.07	1.47
			Na^+ ions (NaCl)	1070.85	31.07	1.35
	N 1s	0.43				
	Al 2p	0.00				
Ta_4AlC_3 MAX Phase	Ta $4f_{7/2}$ ($4f_{5/2}$)	32.15				
	C 1s	33.15				
	O 1s	31.51				
	Al 2p	3.19				

Supplementary Table S2: List of western blotting antibodies used in this study.

**Antibody Name**	**Vendor**	**Catalog Number**	**Dilution**
Anti-HLA-DRα antibody	Santa Cruz Biotechnology	sc-55592	1:200
HRP anti-β-actin antibody	Santa Cruz Biotechnology	sc-47778	1:10,000
HRP anti-mouse secondary	Bio-Rad Laboratories	1706516	1:10,000

Supplementary Table S3: List of flow cytometry antibodies used in this study.

Antibody Name	Clone	Vendor	Catalog Number	Quantity (for 10^6 cells in 100 μL)
PE anti-human CD3	UCHT1	BioLegend	300456	0.5 μg
FITC anti-human CD4	RPA-T4	BioLegend	300506	2 μg
PerCP anti-human IFN-γ	4S.B3	BioLegend	502524	0.5 μg
AF647 anti-human IL-4	8D4-8	BioLegend	500712	0.25 μg
FITC anti-rat CD4	W3/25	BioLegend	201505	0.25 μg
PE anti-rat CD25	OX-39	BioLegend	202105	0.25 μg
PE mouse IgG1κ isotype	MOPC-21	BioLegend	400111	n/a
FITC mouse IgG1κ isotype	MOPC-21	BioLegend	400107	n/a
PerCP mouse IgG1κ isotype	MOPC-21	BioLegend	400147	n/a
AF647 mouse IgG1κ isotype	MOPC-21	BioLegend	400135	n/a

Supplementary Table S4: List of quantitative PCR primers used in this study.

Human Gene	Strand	Sequence (5' to 3')
NF-κB (p65)	*s*	GCT GCA TCC ACA GTT TCC AGA
	as	CCC CAC GCT GCT CTT CTA T
IRF1	*s*	CCT CCA CCT CTG AAG CTA CAA C
	as	CCA TCC ACG TTT GTT GGC TG
TAP1	*s*	TCG TTG TCA GTT ATG CAG CG
	as	AAT GGC CAT CTC CCC AAG AG
HLA-A	*s*	GAG TAT TGG GAC CAG GAG ACA C
	as	CCA CGT CGC AGC CAT ACA TTA
B2M	*s*	GAT GAG TAT GCC TGC CGT GT
	as	CTG CTT ACA TGT CTC GAT CCC A
VCAM-1	*s*	GGA AAT GAC CTT CAT CCC TAC CA
	as	ATC TCT GGG GGC AAC ATT GA
ICAM-1	*s*	AGC TTC GTG TCC TGT ATG GC
	as	TTT TCT GGC CAC GTC CAG TT
PECAM-1	*s*	GCT GAC CCT TCT GCT CTG TT
	as	ATC TGG TGC TGA GGC TTG AC
VE-Cadherin	*s*	CTT CAC CCA GAC CAA GTA CAC A
	as	AAT GGT GAA AGC GTC CTG GT
E-Selectin	*s*	CCG AGC GAG GCT ACA TGA AT
	as	GCA TCG CAT CTC ACA GCT TC
P-Selectin	*s*	CAT CCG CTC ACT GCT TTT GC
	as	AAT CCA TGC TTC CGT GGA CA
FAS Ligand	*s*	CTA CCA GCC AGA TGC ACA CA
	as	CCT TGA GTT GGA CTT GCC TGT
CCL2	*s*	AGA TCT GTG CTG ACC CCA AG
	as	GGA GTT TGG GTT TGC TTG TCC
CXCL9	*s*	GGT GTT CTT TTC CTC TTG GGC
	as	TTC TCA CTA CTG GGG TTC CTT G
CXCL10	*s*	AAG TGG CAT TCA AGG AGT ACC T
	as	GGA CAA AAT TGG CTT GCA GGA
HLA-DRα	*s*	ACT CCG ATC ACC AAT GTA CCT C
	as	ACG TTG GGC TCT CTC AGT TC
CIITA	*s*	CAC CAT CCC ATT CAG TGT CCA
	as	TCC AGC GTG GTT AGT GTC CT
PD-L1	*s*	CCT CTG GCA CAT CCT CCA AAT
	as	GCT GGA TTA CGT CTC CTC CAA
CD86	*s*	CGA CGT TTC CAT CAG CTT GTC
	as	TCC AAG GAA TGT GGT CTG GG
ACTB	*s*	CTT CGC GGG CGA CGA T
	as	CCA CAT AGG AAT CCT TCT GAC C

Supplementary Table S5: List of immunocytochemistry antibodies used in this study**.**

**Antibody Name**	**Vendor**	**Catalog Number**	**Dilution**
Mouse anti-rat CD8a	Cedarlane	CL004AP	1:100
Goat anti-human/mouse/rabbit/rat α-SMA	Novus Biologicals	NB300-978	1:300
Mouse anti-human vWF	abcam	ab201336	1:200
Rabbit anti-human VE cadherin	abcam	ab33168	1:100
AF488 goat anti-mouse secondary	Invitrogen	A-11017	1:500
AF647 goat anti-rabbit secondary	Invitrogen	A-21246	1:500
AF647 goat anti-mouse secondary	Invitrogen	A-21237	1:500
AF647 donkey anti-goat secondary	abcam	ab150135	1:500

Supplementary Equation S1, S2: ***The Beer-Lambert Theory*** describes UV-Visible absorption arising from the surface of $Ta_4C_3T_x$ MQDs and correlates to its optical properties. The novel α for the aqueous colloidal dispersion of this material was presented in the manuscript.

$$\text{Absorbance (A)} = \log(I_o/I) = \alpha * L * c \qquad \text{(Equation 1)}$$

$$\alpha = A / (L * c) \qquad \text{(Equation 2)}$$

where,

I_o is the intensity of the light on the colloidal suspensions
α refers the absorptivity of the absorber or absorption coefficient
L signifies the path length that the light travels through the sample
c assigns to the concentration of the material.
